# Using models and maps to inform Target Product Profiles and Preferred Product Characteristics: the example of *Wolbachia* replacement

**DOI:** 10.12688/gatesopenres.14300.1

**Published:** 2023-05-12

**Authors:** Katie Tiley, Julian Entwistle, Bruce Thomas, Laith Yakob, Oliver Brady

**Affiliations:** 1 Centre for Mathematical Modelling of Infectious Diseases, London School of Hygiene & Tropical Medicine, London, UK; 2 Department of Infectious Disease Epidemiology, Faculty of Epidemiology and Population Health, London School of Hygiene & Tropical Medicine, London, UK; 3 IPM Focus Ltd., Rowland’s Castle, Hampshire, UK; 4 The Arcady Group, Richmond, VA, USA; 5 Department of Disease Control, London School of Hygiene & Tropical Medicine, London, UK

**Keywords:** mosquito, dengue, model, arbovirus, policy, intervention, Wolbachia, cost

## Abstract

*Background*

The global prevalence of diseases transmitted by *Aedes aegypti* mosquitoes, such as dengue, Zika and Yellow Fever, is increasing, but development of promising new mosquito control technologies could reverse this trend. Target Product Profiles (TPPs) and Preferred Product Characteristics (PPCs) documents issued by the World Health Organization can guide the research and development pathways of new products and product combinations transitioning from proof of concept to operational use.

*Methods*

We used high resolution global maps of the case and economic burden of dengue to derive programmatic cost targets to support a TPP for *Wolbachia* replacement. A compartmental entomological model was used to explore how release size, spacing and timing affect replacement speed and acceptability. To support a PPC for a hybrid suppress-then-replace approach we tested whether *Wolbachia* replacement could be achieved faster, more acceptably or at a lower cost if preceded by a mosquito suppression programme.

*Results*

We show how models can reveal trade-offs, identify quantitative thresholds and prioritise areas and intervention strategies for further development. We estimate that for *Wolbachia* replacement to be deployable in enough areas to make major contributions to reducing global dengue burden by 25% (in line with 2030 WHO targets), cost must ultimately be reduced to between $7.63 and $0.24 (USD) per person protected or less. Suppression, particularly interventions that induce mosquito sterility, can reduce the number of *Wolbachia* mosquitoes necessary to achieve fixation by up to 80%. A hybrid approach can also achieve fixation faster and potentially improve acceptability, but may not justify their cost if they require major new investments in suppression technologies.

*Conclusions*

Here we demonstrate the value dedicated modelling can provide for interdisciplinary groups of experts when developing TPPs and PPCs. These models could be used by product developers to prioritise and shape development decisions for new *Wolbachia* replacement products.

## Introduction

The *Aedes aegypti* mosquito is the principal vector of dengue, Zika, yellow fever and chikungunya viruses. Dengue incidence has been rising and the WHO Global Vector Control Response 2017 – 2030 reports an annual 96 million cases, 1.9 million DALYs and 9,110 deaths ^1^. Vaccines are only available for yellow fever and are not currently widely used for dengue, though there are other dengue and chikungunya vaccine candidates in clinical trials ^2, 3^. There are no drugs available to combat these infections and so there is a reliance on prevention through vector control. Effective control of this vector is difficult to achieve and sustain given the mosquito’s high reproductive rate and adaptation to urban habitats, with an egg stage that can survive desiccation and a larval phase that can develop in small, temporary water volumes (e.g., water containers and roof gutters). The rapid growth of cities has also favoured this mosquito ^4^. As a result, existing vector control tools alone have generally been unable to sustainably control *Ae. aegypti* or the diseases it transmits over the long term. A range of novel technologies are under development ^5^, including biocontrol through use of *Wolbachia* spp. for population replacement or reduction/suppression, the release of genetically modified mosquitoes (such as Oxitec's 1 ^st^ generation self-limiting technology (1gSLT) ^6^), and other forms of sterile insect technique (SIT).

Mosquitoes infected with *Wolbachia* show reduced rates of virus dissemination, making them less capable of transmitting arboviruses ^7^. *Wolbachia* infection is also dominantly maternally inherited and leads to inviable progeny when *Wolbachia* males and wild-type females mate. This means that *Wolbachia* can be used to either replace the existing mosquito population with a lower competence phenotype by releasing females (or males and females) or suppress the existing population by releasing only males.

*Wolbachia* population replacement involves regular releases of *Wolbachia*-infected mosquitoes into a wild mosquito population over a period of several months. Once a critical proportion of mosquitoes in the population have *Wolbachia*, coverage will continue to increase to fixation without further releases, but below this threshold *Wolbachia* coverage may decline (possibly to zero) once releases stop due to fitness costs associated with released mosquito strains ^8^. Operationally, the chance and speed of exceeding this threshold and achieving self-sustaining coverage can be achieved by: increasing the number of releases, decreasing the time gap between releases and increasing the ratio of *Wolbachia*-infected *Ae. aegypti* in relation to wild-type *Ae. aegypti* in each release. All of these options increases cost and can also lead to undesirable temporary increases in the *Ae. aegypti* mosquito population that could be a key barrier to community acceptability ^9, 10^.

A growing range of entomological, epidemiological and modelling evidence supports the widespread, long-term effectiveness of *Wolbachia* replacement ^11– 13^. This includes a randomised controlled trial (RCT) of *wMel Wolbachia* in Yogyakarta City, Indonesia which demonstrated a 77% reduction in dengue incidence and an 86% reduction in hospitalizations ^14^. To date, however, *Wolbachia* replacement programmes have only been conducted in specific mid-sized cities or specific neighbourhoods of cities. Thirteen countries have implemented replacement programmes at various levels of scale, with 12 through the World Mosquito Program (WMP) and an independent programme in Malaysia ^15, 16^. Meanwhile, China (with *Ae. albopictus*), Singapore, and the USA have so far chosen to use suppression-based programs due to perceived greater compatibility with their existing intensive and long-term efforts to suppress mosquito populations ^17– 19^. To date, there have been no large-scale (national or regional), non-donor funded implementations of *Wolbachia* replacement or suppression approaches.

These novel technologies ( *Wolbachia* replacement, *Wolbachia* suppression *,* 1gSLT and SIT *)* are subjects of ongoing development, evaluation, demonstration and scale-up in various high-burden programmatic and private settings. In addition, there is the potential to combine *Wolbachia* population replacement with other suppression technologies in ways that may be synergistic from a cost, community acceptance, or dengue incidence reduction perspective ^9^. Specifically, a programme of suppression followed by replacement has the potential to increase the likelihood of successful *Wolbachia* establishment and reduce the cost of, and risk of mosquito exacerbation associated with, achieving establishment ^20^.

Development and transition to scale of new products and strategies can be accelerated by the development of internationally recognised Target Product Profiles (TPPs) and Preferred Product Characteristics (PPCs) documents ^21^. TPPs provide specific quantitative guidance on the key characteristics a product must (minimum target), or should ideally (preferred target), meet when developed into a deployable mass market product. PPCs identify broader areas of unmet need and aim to stimulate new products or product combinations that can address these needs. In early 2022 the WHO convened a Technical Advisory Group (TAG) to develop a draft TPP for *Wolbachia* replacement and a draft PPC for a hybrid mosquito suppression then *Wolbachia* replacement strategy. TAG members agreed that these WHO TPPs and PPCs should closely align with the WHO’s strategy and goals to control dengue globally. As such the WHO’s goal to reduce dengue incidence by 25% by 2030 (2010 – 2020 baseline ^22^) provided a basis to understand the scale and range of settings in which these TPPs, PPCs and the products they ultimately produce are relevant. Computational models can play a key role in the development of TPPs and PPCs due to their ability to generalise beyond areas where data have been collected and make predictions if aspects of the product were to change. Here we describe a dynamic compartmental entomological model and a global geospatial economic model that we developed and used to explore how operational and economic aspects of *Wolbachia* replacement are likely to change once the technology is used at scale. 

## Methods

### Global dengue cost model

The global dengue cost model aims to produce high spatial resolution estimates of the economic costs of dengue that would be averted by *Wolbachia* replacement. These were conservatively estimated to be composed of the direct medical cost of treatment of dengue patients and emergency (outbreak) vector control costs. In the absence of primary data on willingness to pay for *Wolbachia* replacement programmes, these averted costs were assumed to represent an appropriate proxy.

A high resolution (5km × 5km at the equator) map of symptomatic dengue case burden was obtained from Bhatt *et al.* ^23^, which estimates the spatial distribution of the 96 (67 – 196) million episodes estimated to occur each year. An average direct medical cost per symptomatic case (2013 USD) was derived for each country from Shepard *et al.* ^24^, considering the different costs of hospitalised and ambulatory cases and the country-specific distribution of symptomatic cases among these two different treatment settings. Direct medical costs include the costs of specific medicines and staff time required to treat a dengue patient and a portion of infrastructural costs and is the most relevant measure of what governments need to pay to treat cases of dengue illness each year. All costs were inflated from 2013 to 2020 USD using World Bank country GDP deflators with a maximum capped value of a two-fold increase ^25^.

A literature review on the cost of vector control in dengue endemic countries was conducted and identified studies with national and subnational estimates of vector control costs for 17 countries. Twenty studies included costs of routine vector control activities and seven studies included costs of vector control during dengue outbreaks (supplementary file 1 in Data Availability). All vector control cost values were converted back to local currencies using the exchange rate at the time of the costing, inflated to 2020 using country GDP deflators from the World Bank ^25^, and then converted to 2020 US dollars using 2020 exchange rates published by the World Bank ^26^. To make predictions of per capita routine vector control costs for countries without costing data, a Poisson generalized mixed linear model was fit to the costing data with national GDP per capita (log scale) as a covariate and national-level random effects. Predictions were then made for all countries globally using World Bank GDP per capita figures from 2020. For countries where this data was missing (some small Caribbean and Pacific Island nations), global median GDP per capita was assumed. Of the seven studies identified that included costs of vector control during dengue outbreaks, five studies gathered information on both routine and outbreak vector control activities. We assume that implementing a *Wolbachia* release program will not avert routine (principally preventative) vector control costs because *Wolbachia* replacement is unlikely to eliminate dengue in most settings and additional vectors (e.g., *Ae. albopictus*) and nuisance biting mosquitoes will still drive a need for routine vector control activities. Instead, it was assumed that the implementation of *Wolbachia* replacement will significantly limit the size of outbreaks and their required vector control response and thus cost. These studies suggested that during outbreaks, the monthly cost of vector control increases by 20–50%. Three scenarios were explored where additional avertable outbreak costs composed 35% of routine monthly vector control costs for a duration of three months every year, with a sensitivity analysis exploring lower (20%) or higher (50%) values.

Total annual averted costs were estimated assuming *Wolbachia* replacement results in a 70% reduction in symptomatic cases (and their associated costs) and 100% of emergency (outbreak response) vector control costs. This is based on a conservative interpretation of the 77% effectiveness of *wMel Wolbachia* measured in the Yogyakarta trial ^14^ and the expectation of variable effectiveness across areas with different transmission intensities ^13^. While *wMel Wolbachia* replacement has been shown to be stable in *Aedes* mosquito populations for over ten years in Australia ^12^, it is unclear how many future years of averted dengue costs would be appropriate to consider when estimating government or other funder willingness to pay. We therefore estimate total averted costs for three-, five- and ten-year time horizons and assume that these costs represent the maximum price a government or funder would be willing to pay for *Wolbachia* replacement in a given setting. To quantify uncertainty around these thresholds, the analysis was repeated with values from the upper and lower bounds of the case burden ^23^ and economic burden ^24^ estimates and with 50% and 20% avertable vector control outbreak proportions respectively.

*Wolbachia* replacement is most cost effective if targeted to higher density, high dengue burden areas ^27^. We therefore identified which areas (5km × 5km pixels) would need to be targeted to reach the WHO goal of reducing global dengue burden by 25% in the most net cost-efficient manner. To do this, each 5km × 5km pixel was ranked from highest to lowest based on a benefit (averted medical and outbreak costs) to cost (approximate *Wolbachia* programme cost estimate based on population density from Brady *et al.* ^27^) ratio. Cumulative averted cases were then calculated and pixel selection ended when averted cases first exceeded 25% of the global total. The averted costs in the last, least cost-efficient pixel included in this subset then gave the cost threshold for *Wolbachia* replacement programmes, i.e., if *Wolbachia* replacement can be achieved at this cost (or lower) it will be possible, from the cost-efficacy perspective, to implement the intervention in enough areas to reduce the global burden of dengue by 25%. An alternative scenario was also calculated where it was assumed that *Wolbachia* replacement is only required to account for half of this global target, i.e., a 12.5% global burden reduction. Because such a global targeting approach prioritises countries with higher GDP, we also calculated a scenario where 95% of dengue endemic countries (defined as >10,000 symptomatic dengue infections a year as estimated by Bhatt *et al.* ^23^) needed to achieve at least a 25% burden reduction through deploying *Wolbachia* replacement, to explore the cost threshold implication of a much wider deployment with improved equity between countries ^28^.

### Entomological model overview

This compartmental mechanistic model follows *Aedes aegypti* population dynamics at egg, larvae, pupae, and adult stages, with pupae developing into female and male adults in equal proportion and each stage subject to a constant death rate:


$$\;\begin{array}{c} \frac{dO}{dt}=\varphi F-\alpha_{O}O-\mu_{O}O\\ \frac{dL}{dt}=\alpha_{O}O-\alpha_{L}L-\mu_{L}L\\ \frac{dP}{dt}=\alpha_{L}\frac{L}{1+{\left(\gamma L\right)}^{\beta }}-\alpha_{P}P-\mu_{P}P\\ \frac{dM}{dt}=0.5\alpha_{P}P-\mu_{M}M\\ \frac{dF}{dt}=0.5\alpha_{P}P-\mu_{F}F \end{array}\;(1)$$

*O* denotes the number of eggs, *L* larvae, *P* pupae, *M* adult males, and *F* adult females. *φ* is the daily egg-laying rate of adult females. *α _O_* denotes the rate at which eggs develop into larvae and the *μ _O_* death rate of eggs. Similarly, *α _L_* denotes the rate at which larvae develop into pupae and *μ _L_* the larval death rate and *α _P_* denotes the rate at which pupae develop into adults and *μ _P_* the pupal death rate. *μ _M_* and *μ _F_* are the adult male and female death rates, respectively. Survival of larvae to pupal stage is density dependent, and using the flexible formulation proposed by Maynard Smith and Slatkin ^29^, includes the parameter *γ* which determines the density at which mortality remains proportionate and the parameter *β* the ‘abruptness’ of density-dependence. 

These equations were then further developed to account for *Wolbachia* deployments, respectively impacting mating and larval survival:


$$\begin{array}{c} \frac{dO}{dt}=\varphi F\frac{M+c_{i}M_{W}}{M+M_{W}}-\alpha_{O}O-\mu_{O}O\\ \frac{dP}{dt}=\alpha_{L}\frac{L}{1+{\left(\gamma (L+L_{W})\right)}^{\beta }}-\alpha_{P}P-\mu_{P}P \end{array}$$

Where, *c _i_* denotes the failure rate of cytoplasmic incompatibility for the *Wolbachia*-infected adult males ( *M _W_*), and *Wolbachia*-infected larvae in the wild ( *L _W_*) also contribute towards larval competition.

While it is important that all wild-hatched larvae are subject to the same density dependence as they are occupying the same habitat, the introduced *Wolbachia*-infected eggs will be released in their own distinct larval habitat (self-contained release containers), therefore their survival is not impacted by the densities of wild-hatched larvae. The equations of released *Wolbachia* ( *rW*) are as follows:


$$\begin{array}{l} \begin{array}{c} \frac{dO_{rW}}{dt}=RR(\dot{F}+\dot{M})-\alpha_{O}O_{rW}-\mu_{O}O_{rW}\\ \frac{dL_{rW}}{dt}=\alpha_{O}O_{rW}-\alpha_{L}L_{rW}-\mu_{L}L_{rW} \end{array}\\ \frac{dP_{rW}}{dt}=\alpha_{L}\frac{L_{rW}}{1+{\left(\gamma L_{rW}\right)}^{\beta }}-\alpha_{P}P_{rW}-\mu_{P}P_{rW} \end{array}\;(2)$$

*O _rW_* denotes the number of released *Wolbachia*-infected eggs, which is the product of the release ratio ( *RR*) and the equilibrial adult population prior to control ( *Ḟ* + *Ṁ*). *L _rW_* denotes the number of *Wolbachia*-infected larvae resulting from released eggs, and *P _rW_* denotes the number of *Wolbachia*-infected pupae resulting from released eggs. The aquatic-stage *Wolbachia*-infected *Ae. aegypti* that hatch outside of the release containers (subscript ‘ *W*’ instead of ‘ *rW*’) are tracked separately from those which are newly released. The wild-hatching *Wolbachia*-infected mosquitoes follow these dynamics:


$$\begin{array}{c} \frac{dO_{W}}{dt}=\varphi F_{W}-\alpha_{O}O_{W}-\mu_{O}O_{W}\\ \frac{dL_{W}}{dt}=\alpha_{O}O_{W}-\alpha_{L}L_{W}-\mu_{L}L_{W}\\ \frac{dP_{W}}{dt}=\alpha_{L}\frac{L_{W}}{1+{\left(\gamma (L+L_{W})\right)}^{\beta }}-\alpha_{P}P_{W}-\mu_{P}P_{W} \end{array}\;(3)$$

*Wolbachia*-infected adult mosquitoes comprise those that have emerged from the wild combined with those emerging from release containers:


$$\;\begin{array}{c} \frac{dM_{W}}{dt}=0.5\alpha_{P}(P_{W}+P_{rW})-\mu_{M}\varepsilon M_{W}\\ \frac{dF_{W}}{dt}=0.5\alpha_{P}(P_{W}+P_{rW})-\mu_{F}\varepsilon M_{W} \end{array}\;(4)$$

*ε* denotes the relative mortality of *Wolbachia*-infected adult mosquitoes compared to uninfected. A sensitivity analysis explored the impact of *Wolbachia* infection fitness costs on mosquito population dynamics under *Wolbachia* release scenarios by varying *ε* (Supplementary Figure 1). Parameter definitions and values are shown in Table 1.

**Table 1.** Model parameters values.

Parameter	Description	Value	Reference
*φ*	Daily egg laying rate of adult females	500*(1/14)	Otero *et al.*, 2006 ^30^
*M _null_*	Male uninfected adults	*M* + *c _i_M _w_*	-
*F _all_*	Total female adults	1+ *F* + *F _w_*	-
*M _all_*	Total male adults	1 + *M* + *M _W_* + *c _M_M _s_*	-
*α _O_*	Daily rate eggs hatch into larvae	0.5	Marinho *et al.*, 2016 ^31^
*α _L_*	Daily rate larvae develop into pupae	0.18	Marinho *et al.*, 2016 ^31^
*α _P_*	Daily rate pupae develop into adults	1	Masters *et al.*, 2020 ^32^
*μ _O_*	Daily mortality rate of eggs	0.01	Trpis, 1972 ^33^
*μ _L_*	Daily mortality rate of larvae	0.1*	Couret *et al.*, 2014 ^34^
*μ _P_*	Daily mortality rate of pupae	0.1*	Couret *et al.*, 2014 ^34^
*μ _M_*	Daily mortality rate of adult males	1/14	Yakob *et al.*, 2008 ^10^
*μ _F_*	Daily mortality rate of adult females	1/14	Yakob *et al.*, 2008 ^10^
*γ*	Determines the density at which mortality remains proportionate	1	Bellows, 1981 ^35^
*β*	Determines the ‘abruptness’ of density dependence	0.5	Bellows, 1981 ^35^
RR	Release ratio of *Wolbachia*-infected adults compared to total adult mosquitoes	Variable	Estimated
*c _i_*	Proportion of cytoplasmic incompatibility that fails	0.012	Walker *et al.*, 2011 ^7^
*c _m_*	Competitiveness of released sterilised males	0.5	Winskill *et al.*, 2014 ^36^
*c _v_*	Proportion of adult population reached by adulticide	0.141	Estimated, described in adulticide section
*ε*	Relative mortality of *Wolbachia*-infected adult mosquitoes compared to uninfected	1.2	Joubert *et al.* 2016 ^37^
s	Adjustment parameter which matches average seasonal mosquito population to non-seasonal equilibrium mosquito population	2.09	Estimated, described in seasonality section

### Suppression

*Wolbachia*-infected egg release was also explored after first deploying suppression interventions. The suppression techniques analysed were the release of 1 ^st^ generation self-limiting technology (1gSLT), sterile insect technique (SIT), Male *Wolbachia* release, environmental management, larvicides, and adulticides. Each type of suppression was included as a function of time, *t*, so that a value which influences model dynamics is pulsed at specific times or maintained over a specific period. The efficacy of each method was based on evidence sourced from the literature, selected with a preference for large randomised-controlled trials, however, each suppression method works differently and trials to measure effectiveness vary with study design. Efficacy of a single burst of application was preferable but only found for adulticide. Studies measuring repeated concurrent applications of 1gSLT and Male *Wolbachia* release, environmental management and larvicides used an interrupted time series design. No suitable publications were found giving field estimates of effectiveness for SIT. The impact of variations in study design are discussed in more detail below.

***1gSLT.*** Release of 1gSLT adult males produce offspring with wild females of which only the males develop to adulthood from the pupal stage. 1gSLT was included in the model by pulsing adult males into a sterile adult male compartment *M _S_* weekly, which then contributed to the production of sterile eggs, *O _S_*, which developed through sterile larval and pupal compartments, *L _S_* and *P _S_*, contributing to a density dependant survival function. *RR _supp_* denotes the release ratio for mosquito release suppression techniques. *ε* denotes the relative mortality of *Wolbachia*-infected adult mosquitoes compared to uninfected adult mosquitoes.


$$\begin{array}{c} \frac{dO_{S}}{dt}=\frac{\varphi F_{all}c_{M}M_{S}}{M_{all}}-\alpha_{O}O_{S}-\mu_{O}O_{S}\\ \frac{dL_{S}}{dt}=\alpha_{O}O_{S}-\alpha_{L}L_{S}-\mu_{L}L_{S}\\ \frac{dP_{S}}{dt}=\alpha_{L}\frac{L_{S}}{1+{\left(\gamma (L+L_{w}+L_{S})\right)}^{\beta }}-\alpha_{P}P_{S}-\mu_{P}P_{S}\\ \frac{dM_{S}}{dt}=RR_{supp}f_{RIDL}-\mu_{M}\varepsilon M_{S} \end{array}\;(5)$$

Parameters for the fixed rate efficacy of 20%, 50%, and 80% were calculated by comparing the total adult population at model equilibrium with the minimum adult population reached after five weeks of application. The literature-derived efficacy values were 45% five weeks after the last suppression period and 70% ten weeks after the last suppression period ^38^, calculated by comparing the total adult population at model equilibrium to the total adult population after five- or ten-weeks of suppression which achieved the desired efficacy (summarised in Supplementary Table 1). A caveat of this approach is that the resulting minimum adult population is reached later than five- or ten-weeks, therefore, the maximum efficacy calculated in these scenarios is marginally greater than the literature value stated (shown in Supplementary Figure 2).

***SIT.*** SIT involves releasing sterile adult males which produce sterile eggs (in the same manner of equation 5) that do not develop further. SIT was included in the model by pulsing adult males into the sterile adult male compartment, *M _S_*, which then contributed to the production of sterile eggs which then do not develop further.


$$\;\frac{dM_{S}}{dt}=RR_{supp}f_{SIT}0.5\alpha_{P}P_{S}-\mu_{M}\varepsilon M_{S}\;(6)$$

Parameters for the fixed rate efficacy of 20%, 50%, and 80% were calculated by comparing the total adult population at model equilibrium with the minimum adult population reached after five weeks of application. There are currently no published randomised controlled trials for suppression of *Aedes* mosquitoes using SIT, therefore 50% efficacy is shown for SIT in analyses where only literature-derived efficacy is used.

***Male Wolbachia release*.** Male *Wolbachia* release involves releasing only *Wolbachia*-infected adult males, resulting in no offspring due to cytoplasmic incompatibility with the local non- *Wolbachia*-infected females. Male *Wolbachia* release was implemented in the model by pulsing *Wolbachia*-infected adult males into the *Wolbachia*-infected male compartment:


$$\frac{dM_{W}}{dt}=RR_{supp}f_{IIT}0.5\alpha_{P}(P_{W}+P_{rW})-\mu_{M}\varepsilon M_{W}\;(7)$$

Parameters for the fixed rate efficacy of 20%, 50%, and 80% were calculated by comparing the total adult population at model equilibrium with the minimum adult population reached after five weeks of application. The literature-derived efficacy values were 60% five weeks after the last suppression period and 95% ten weeks after the last suppression period ^39^, calculated by comparing the total adult population at model equilibrium to the total adult population after 5- or 10-weeks of suppression which achieved the desired efficacy (summarised in Supplementary Table 1). Similar to 1gSLT, a caveat of this approach is that the resulting minimum adult population is reached later than 5- or 10-weeks, therefore, the maximum efficacy calculated in these scenarios is marginally greater than the literature value stated (shown in Supplementary Figure 2).

***Environmental management.*** Environmental management reduces the amount of egg-laying habitat which was simulated by manipulating the egg production rate:


$$\begin{array}{c} \frac{dO}{dt}=f_{EM}\varphi F\frac{M_{null}}{M_{all}}-\alpha_{O}O-\mu_{O}O\\ \frac{dO_{W}}{dt}=f_{EM}\varphi F_{W}\frac{M+M_{W}}{M_{all}}-\alpha_{O}O_{W}-\mu_{O}O_{W} \end{array}\;(8)$$

Parameters for the fixed rate efficacy of 20%, 50%, and 80%, and the literature-derived efficacy of 47.4% ^40^, were calculated by comparing the total adult population at model equilibrium with the new population equilibrium reached after applying this technique for the duration of the simulation; this emulates real-world scenarios in which application and outcome are typically long-term (summarised in Supplementary Table 1).

***Larvicides.*** Larvicides were simulated using the example of guppies, reducing the number of larvae through increased mortality to all aquatic life stages of both uninfected and wild-hatching *Wolbachia*-infected mosquitoes.


$$\begin{array}{c} \frac{dO}{dt}=\varphi F\frac{M_{null}}{M_{all}}-\alpha_{O}O-f_{LV}\mu_{O}O\\ \frac{dO_{W}}{dt}=\varphi F_{W}\frac{M+M_{W}}{M_{all}}-\alpha_{O}O_{W}-f_{LV}\mu_{O}O_{W}\\ \frac{dL}{dt}=\alpha_{O}O-\alpha_{L}L-f_{LV}\mu_{L}L\\ \frac{dL_{W}}{dt}=\alpha_{O}O_{W}-\alpha_{L}L_{W}-f_{LV}\mu_{L}L_{W}\\ \frac{dP}{dt}=\alpha_{L}\frac{L}{1+\gamma {\left(L+L_{W}+L_{S}\right)}^{\beta }}-\alpha_{P}P-f_{LV}\mu_{P}P\\ \frac{dP_{W}}{dt}=\alpha_{L}\frac{L_{w}}{1+{\left(\gamma (L+L_{w}+L_{S})\right)}^{\beta }}-\alpha_{P}P_{W}-f_{LV}\mu_{P}P_{W} \end{array}\;(9)$$

Parameters for the fixed rate efficacy of 20%, 50%, and 80%, and the literature-derived efficacy of 44% ^41^, were calculated by comparing the total pupae population at model equilibrium with the new pupae equilibrium reached after applying this technique for the duration of the simulation; similar to environmental management techniques this emulates real-world scenarios in which application and outcome are typically long-term (summarised in Supplementary Table 1).

***Adulticides.*** Finally, the deployment of adulticides through fogging and chemical spraying were simulated by pulses which manipulate the mortality rates of adult male and female compartments:


$$\begin{array}{c} \frac{dM}{dt}=0.5\alpha_{P}P-\mu_{M}M-f_{AD}c_{v}M\\ \frac{dM_{W}}{dt}=0.5\alpha_{P}(P_{W}+P_{rW})-\mu_{M}\varepsilon M_{W}-f_{AD}c_{v}M_{W}\\ \frac{dF}{dt}=0.5\alpha_{P}P-\mu_{F}F-f_{AD}c_{v}F\\ \frac{dF_{W}}{dt}=0.5\alpha_{P}(P_{W}+P_{rW})-\mu_{F}\varepsilon F_{W}-f_{AD}c_{v}F_{W} \end{array}\;(10)$$

Parameters for the fixed rate efficacy of 20%, 50%, and 80% were calculated by comparing the total adult population at model equilibrium with the minimum adult population after one application. Mani *et al.* ^42^ reported an initial 94% reduction in mosquito resting density from application of deltacide, a synergized mixture of pyrethroids, after which the population completely recovered within seven days (summarised in Supplementary Table 1). This combination of great suppression and swift recovery could not be replicated in the model by only manipulating adult mortality; this may be because some portion of the reduction in resting density was due to a repellent effect, which has been noted as a possibility by the source paper ^42^ or because recovery was due to recolonisation by neighbouring populations which is not modelled here. To fit this literature efficacy a parameter for 94% mortality rate was first calculated by comparing the total adult population at model equilibrium with the minimum adult population after one application and subsequently a coverage parameter, denoted as *c _v_*, was fitted using the 94% efficacy parameter. The highest proportion of coverage was calculated which allowed 80% population recovery within three weeks of suppression using literature-derived efficacy; the assumption of this recovery speed was explored with a sensitivity analysis, shown in Supplementary Figure 3, and found to be minimally affected by changing the number of weeks taken for recovery.

### Seasonality

Seasonality is defined by using a normalised and smoothed lowess curve of average monthly precipitation (sourced from www.meteoblue.com) to create a score, bounded by 0 and 1, for a seasonality profile with a distinct wet and dry season each year, produced using data from Rio de Janeiro. Within the model, this score influences *γ* within the density dependent function. The density-dependent seasonal function of larval survival is:


$$\;\frac{L}{1+{\left(\gamma f_{K}s(L+L_{w}+L_{S})\right)}^{\beta }}\;(11)$$

The seasonality function, *f _K_*, returns a precipitation score dependent on time, *t*, which affects the rate larvae develop and enter the pupal stage. *s* is a constant, calculated to ensure the average mosquito population in the seasonal model is within 0.5 of the non-seasonal model equilibrium which allows comparability to the non-seasonal analyses as suppression efficacy parameters and functions execute according to this average. This affects all wild-hatched model compartments; however, the released *Wolbachia*-infected eggs are introduced in containers which isolate them from the limits of rainfall dependent egg hatching and larval growth (seasonal population dynamics shown in Supplementary Figure 4). Placement of the seasonality function within the model was explored (Supplementary Table 2) in addition to the impact of temperature, rather than precipitation, on larval development (Supplementary Figure 5).

### Release scenario analyses

*Wolbachia* coverage data was extracted from the report by Utarini *et al.* ^14^ using WebPlotDigitzer ^43^ and the intervention cluster-level results used as reference to calibrate the model. Simulations of *Ae. aegpti* population dynamics were undertaken to investigate the intervention conditions which would produce the desired *Wolbachia* coverage levels (>95%) in the mosquito population. Specifically, a range of release ratios (0.03 to 0.1, in increments of 0.01) and number of releases (9 to 14, pulsed every 14 days) were explored and their influence on the number of days until target coverage was achieved.

The influence of suppression efficacy (20%, 50%, 80%, and a literature derived efficacy) and the week of switch from suppression to *Wolbachia* release (1 to 10) was investigated in terms of the minimum release ratio (0.0025 to 0.4, explored in increments of 0.0025) necessary to reach *Wolbachia* target coverage within six months of the first *Wolbachia* release.

The seasonality model was run for 18 months and the initial six months burn in period needed for model calibration was discarded; five weeks of suppression followed by five rounds of *Wolbachia* replacement release were simulated, exploring minimum RR (0.0025 to 0.4 in increments of 0.0025) required to reach target coverage within six months of first *Wolbachia* replacement release.

## Results

### Exploring the sensitivity of *Wolbachia* replacement to key release characteristics

To explore how self-sustaining *Wolbachia* replacement can best be achieved and its sensitivity to various operational parameters we formulate, fit and simulate from an entomological dynamic compartmental model. By calibrating the release ratio parameter, our model showed a good fit to the mosquito release data from the Yogyakarta RCT with replacement dynamics and coverage levels proceeding at a similar rate. The model reached 50% coverage after 121 days, compared to an average of 117 days observed in the RCT, and 90% coverage after 180 days, compared to an average of 239 days observed in the RCT (Supplementary Figure 6). As *Wolbachia* reaches fixation the model slightly overestimates final *Wolbachia* coverage, likely due to prevalence being suppressed in the RCT due to migration of uninfected adult mosquitoes from outside the release area which were not included in our model and would be reduced when implemented as a wide-scale blanket intervention.

**Figure 1.**
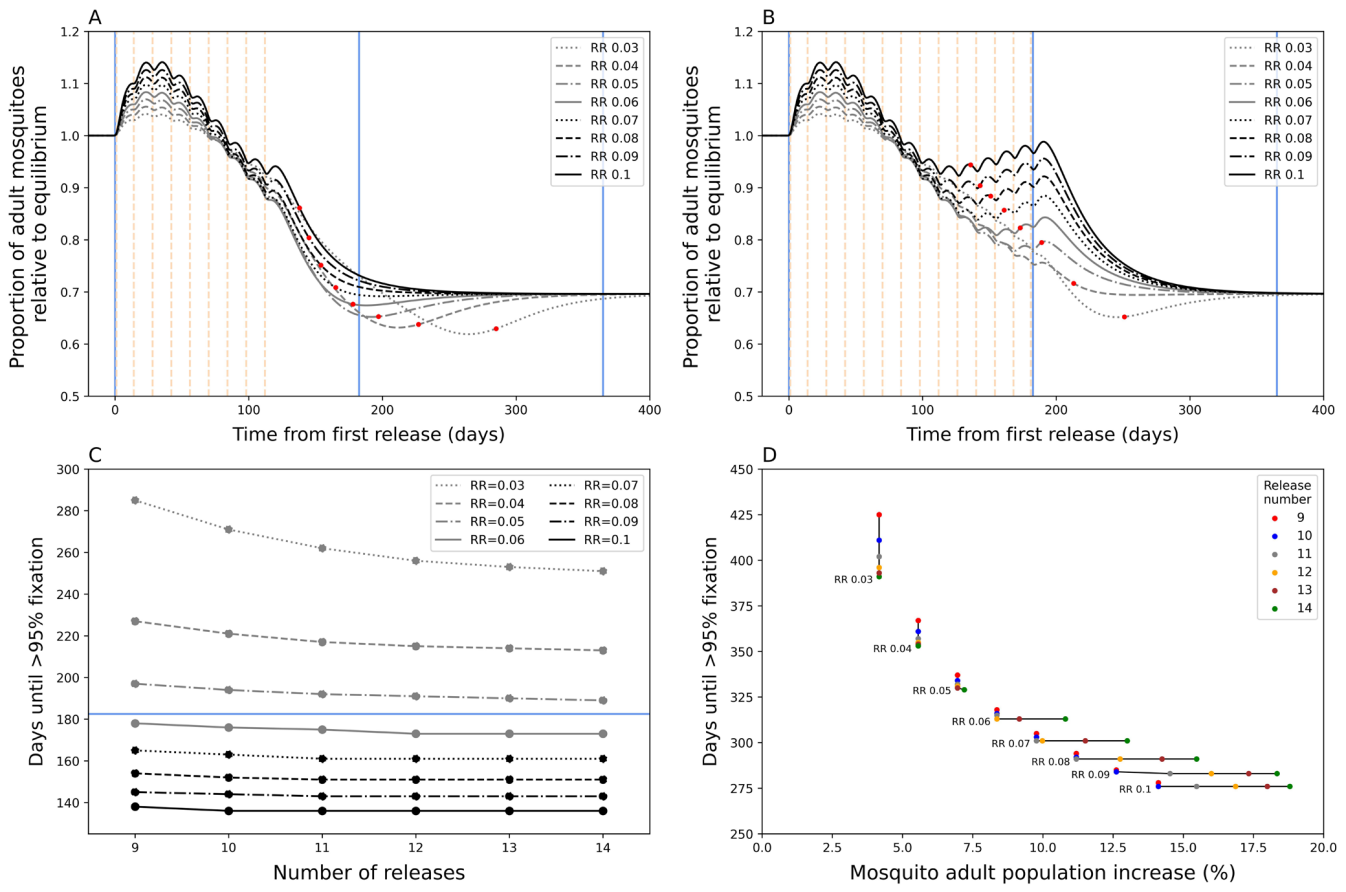
Mosquito dynamics of *Wolbachia* replacement. Figures **A** and **B** show the total adult mosquito population size over time during *Wolbachia* replacement after a nine ( **A**) and fourteen ( **B**) release (dotted vertical lines) round programme. Red dots indicate the date at which target coverage (>= 95% *Wolbachia* coverage) was first achieved. The blue lines show the time points from first release at 0 days, 6 months, and 1 year from left to right. **C**) Days until *Wolbachia*-infected adult mosquitoes reach target coverage for different numbers of releases and release ratios (RR). The horizontal blue line indicates 6 months. **D**) The percentage increase in total mosquito population for different numbers of releases and release ratios. This is calculated as the sum of the peak increase after initial release compared to the pre-release population equilibrium and the peak increase after target coverage is achieved compared to post-fixation equilibrium population, the latter excludes lower RR instances where the population at fixation is lower than the new population equilibrium.

During a *Wolbachia* replacement programme, our model predicts that the total adult mosquito population experiences a temporary exacerbation above baseline levels, followed by a decline before reaching a new equilibrium once *Wolbachia* fixation has been achieved ( Figure 1A, 1B). Due to the fitness cost of *Wolbachia* (conservatively assumed to be 20% ^37^, but variable depending on environment), this new equilibrium mosquito population size is predicted to be lower than before *Wolbachia* release.

As expected, *Wolbachia* target coverage can be achieved faster by increasing RR and/or by increasing the number of releases ( Figure 1C). Our model shows that increasing RR of each release will reduce time to target coverage more than increasing the number of release rounds, particularly at higher RR values. Above a RR of 0.06, increasing the number of release rounds has little additional effect on time to target coverage. Our model predicts that achieving *Wolbachia* target coverage within six months of the first release is possible with RR >= 0.06 with >= 9 releases. Higher RR and number of release rounds lead to ever diminishing decreases in time to target coverage. Increasing the RR from 0.06 to 0.1 is only predicted to increase time to target coverage by 40 days (nine release programme, Figure 1C).

Higher RRs and higher release round numbers also lead to disproportionately undesirable temporary exacerbation issues, particularly at higher values ( Figure 1A,B,D). A doubling of RR from 0.05 to 0.1 could lead to an approximate doubling of exacerbation (6.96% to 14.11%) under a nine-release programme, but this could be up to 2.6 times more (7.20% to 18.80%) if the number of release rounds were increased to 14. This is because prolonged releases at high RR led to a secondary peak in mosquito abundance that prolongs the period of exacerbation ( Figure 1B); in the fourteen-release programme this is still less than the original population, however, increasing number of releases could expect this secondary peak to eventually exceed the prior population size.

Overall, these simulations suggest the importance of balancing speed of *Wolbachia* replacement with the potentially negative consequences of temporarily exacerbating the mosquito population. In combination with other field evidence, this work supported the TPP’s guidance on “time to achieve target coverage”. The models suggested that a time to achieve coverage of less than 12 months was highly feasible (minimum TPP standard) and that a goal of 6 months (preferred TPP standard) was achievable. To counterbalance the issue of exacerbation, the TPP included a criterion for “community acceptability” that states that any increase in nuisance biting through the chosen release characteristics is “acceptable to local residents”, recognising that the definition of “acceptable” is likely to be highly context specific.

### Global cost targets for *Wolbachia* replacement

The TAG identified cost as a key reason limiting wider adoption of *Wolbachia* replacement and therefore a “mature product cost once implemented at scale” criterion was a key feature of the TPP. This cost criterion needed to be low enough to drive innovation and ensure a significant proportion of the global population at risk of dengue can benefit, but not too low as to exclude promising products from further development.

Because detailed data on willingness to pay was unavailable at the time of analysis, we developed a range of scenarios that assume willingness to pay is approximated by the costs of treating dengue cases and of vector control in response to outbreaks over a range of years ( Table 2). Each scenario gave a theoretical cost each 5km × 5km area would be willing to pay for *Wolbachia* replacement. Areas that supported higher costs typically had higher dengue burden but were also heavily influenced by the cost of dengue treatment and prevention.

**Table 2.** The predicted target cost per person for *Wolbachia* replacement based on different assumptions about desired global impact (rows) and averted medical and outbreak control costs (assumed proxy of willingness to pay, columns). *Wolbachia* replacement would need to be at or below this cost to achieve each impact scenario in full. All values show median estimates in 2020 US dollars, brackets show model predicted uncertainty around the true value of this cost threshold at the 95% credible interval level).

Impact scenario	Required cost per person covered (10 years benefit)	Required cost per person covered (5 years benefit)	Required cost per person covered (3 years benefit)
12.5% global burden reduction	$7.63 (5.15 – 29.42)	$4.10 (2.77 – 15.83)	$2.54 (1.71 – 9.78)
25% global burden reduction	$4.33 (2.73 – 18.95)	$2.33 (1.47 – 10.20)	$1.44 (0.91 – 6.30)
12.5% national burden reduction	$0.98 (0.64 – 3.78)	$0.53 (0.34 – 2.03)	$0.33 (0.21 – 1.26)
25% national burden reduction	$0.72 (0.26 – 1.66)	$0.39 (0.14 – 0.89)	$0.24 (0.09 – 0.55)

**Figure 2.**
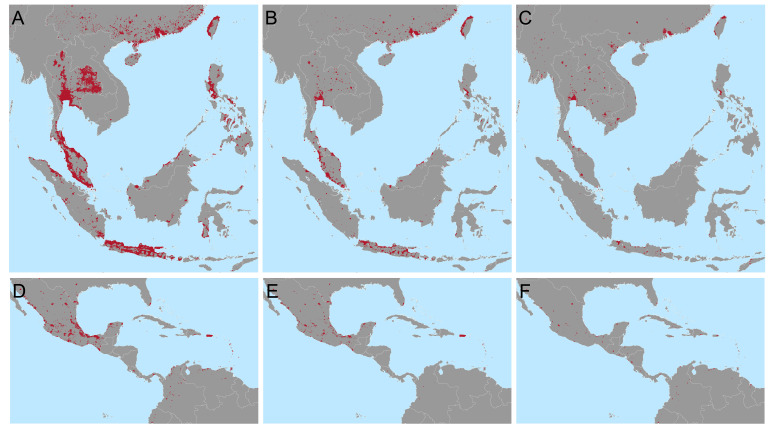
Targeting areas for *Wolbachia* replacement to meet different global and national goals in Southeast Asia ( **A**– **C**) and Central America and the Caribbean **D**– **F**). Maps show the areas most cost efficient to target (red) to reduce the global burden of dengue by 25% ( **A** and **D**) or 12.5% ( **B** and **E**) or the national burden by 25% ( **C** and **F**) based on the cost of treatment and prevention of current dengue burden. Predictions for other areas and lists of municipalities to be targeted are included in the following repository https://github.com/katietiley/Wolbachia_TPP_PPC.git.

**Figure 3.**
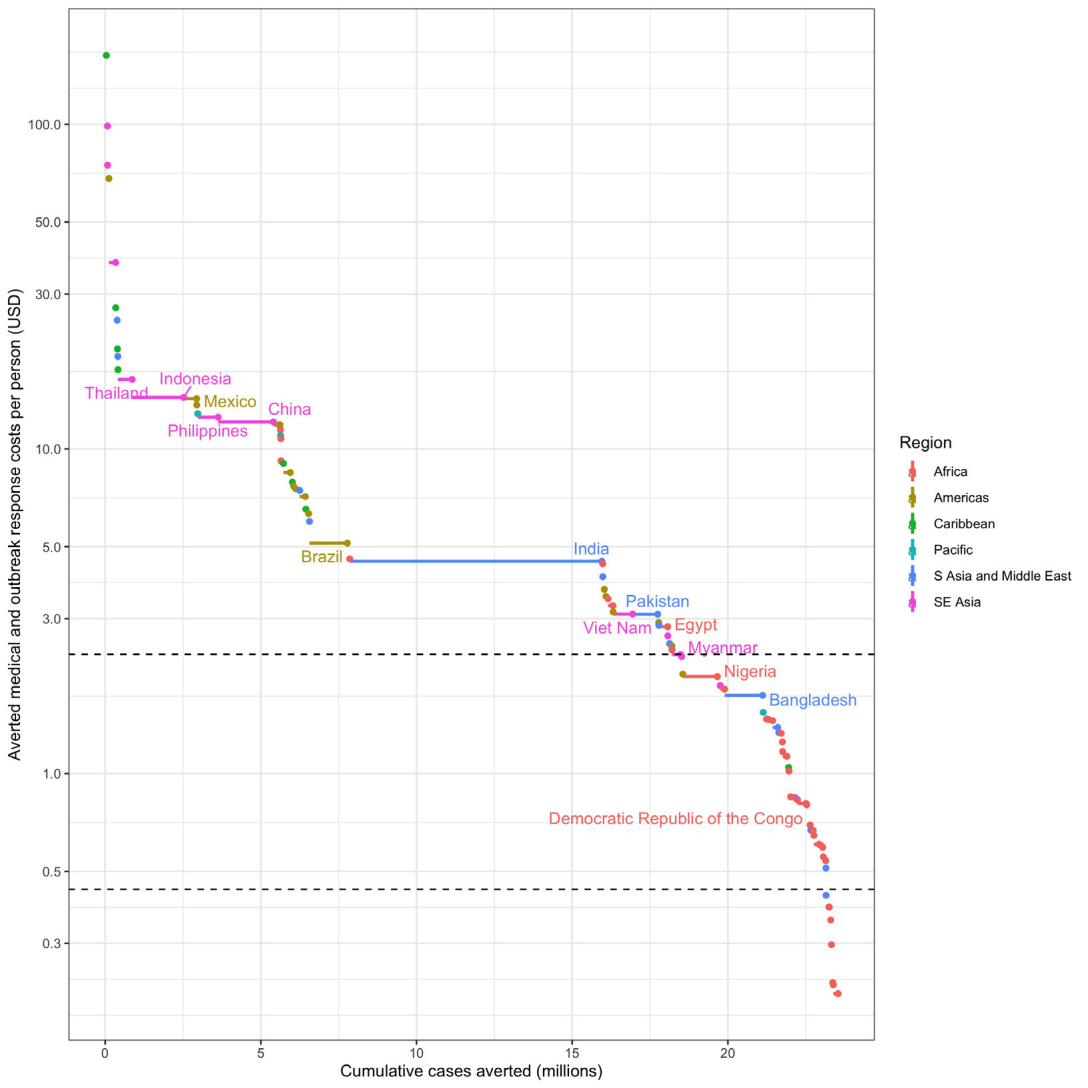
The cumulative contribution of each dengue endemic country to the 25% global reduction in dengue burden and the averted costs (willingness to pay proxy) per person covered at which it can be achieved. Horizontal dotted lines show the cost thresholds of $2.33 per person and $0.24 per person chosen for the TPP minimum and preferred criteria respectively. Only high burden countries are labelled.

We estimate that to achieve a 25% reduction in the global burden of dengue, as per the WHO 2020-2030 goals, using only *Wolbachia* replacement targeted to the most cost-efficient areas, would require releases across 924,557km ^2^ in 73 countries ( Figure 3). This corresponds to 34.7% of the urban (> 300 people per km ^2^) area at risk and just 1.7% of the total area at risk of dengue. If *Wolbachia* only needs to achieve half of the global 25% reduction, with other interventions responsible for the remaining half, *Wolbachia* releases would only need to be targeted to 255,459km ^2^ over 47 countries ( Figure 2B and 2E). However, because these cost estimates are uncertain and because this approach prioritises high income countries where dengue treatment costs are high, we also include a third and fourth targeting scenario where 25% or 12.5% of the national burden must be reduced for the majority (95%) of dengue endemic countries ( Figure 2C and 2F). These scenarios improve equity over dengue-endemic regions. A full list of 2 ^nd^ administrative units included under each targeting scenario, the costs each will support and additional contextual information (population, density, etc) is included in the following repository: https://github.com/katietiley/Wolbachia_TPP_PPC.git.

For *Wolbachia* replacement to be implemented in enough areas to meet these impact targets, the cost of implementation must ultimately be reduced to between $7.63 and $0.24 per person covered depending on scenario ( Table 2). The cost thresholds identified in Table 2 represent the area with the lowest averted costs (assumed lowest willingness to pay) within the areas needed to reach each impact target. This means that many eligible areas, or even whole countries, could support higher programme costs, but ultimately *Wolbachia* replacement will need to be implemented at or below this cost threshold in order to reach the impact target. The distribution of these costs and benefits by country is shown in Figure 3 for the 25% global burden reduction impact target. This shows that while globally *Wolbachia* replacement will need to be achieved for $2.33 per person to meet the 25% impact goal, many countries could support higher costs with many high burden countries able to implement in a wide range of high burden areas above the $10 per person line.

Cost targets become lower (increasingly more ambitious for product development) as impact scenarios become more ambitious or, to a lesser extent, as the accepted duration of benefits becomes shorter (rows and columns in Table 2 respectively). To achieve a 25% national burden reduction in all dengue endemic countries would require a cost target ~ 10x lower than to achieve a 12.5% global dengue burden reduction, emphasising that even higher cost products could still have substantial global impact, but would be less equitable unless subsidised for countries with lower financial capacity for dengue prevention and treatment. Due to high uncertainty in estimates of the true burden of dengue ^23^ and its costs of treatment ^24^ and prevention, uncertainty around these cost thresholds is moderate with higher uncertainty around higher median cost thresholds. 

Each of these scenarios and their respective cost targets were presented to the TAG for discussion and selection for the TPP. Recognising that the TPP minimum criteria should reflect the minimum cost for a product to be viable at substantial scale, TAG members selected the $2.33 per person covered target (corresponding to a 25% global burden reduction with five years of benefit, Table 2). This cost needs to include the programme of activities required to reach 90% coverage of *Wolbachia* in the release areas one year after starting releases. The TPP also makes allowances for a slower programme where 90% coverage is achieved over three years, but this must be achieved at a more stringent minimum TPP cost target of $1.44 per person covered (corresponding to a 25% global burden reduction with three years of benefit, Table 2). However, to challenge developers to meet the more equitable 25% national dengue burden reduction, the TPP preferred cost threshold was set at $0.24 per person covered (corresponding to three years of benefits in this scenario, Table 2). These decisions were also informed by evidence that the current World Mosquito Program cost base for *wMel Wolbachia* replacement is in the US$5-22 per person range ^27^ with a medium-term goal of achieving *Wolbachia* replacement for $1 per person ^44^. Given that these TPP targets represent the lowest averted medical and outbreak control costs per person among all areas where releases are required, there are many areas that could support higher programme costs. Therefore, provided developers can demonstrate the prospect of achieving the TPP cost targets in future, there is scope to operate higher cost programmes before these targets are achieved.

### Exploring the development of a hybrid “suppress then replace” approach

In addition to the draft TPP for *Wolbachia* replacement, the TAG was also tasked to develop a draft PPC for a hybrid mosquito population suppression followed by *Wolbachia* replacement approach. Reducing the natural mosquito population size allows *Wolbachia* replacement programmes to achieve higher release ratios or achieve comparable release ratios by releasing fewer *Wolbachia* mosquitoes. To support the development of the PPC we developed a compartmental entomological model and simulated hybrid strategies with a range of suppression types to answer the questions: can hybrid strategies achieve coverage faster, improve community acceptance and reduce costs relative to a *Wolbachia* replacement programme alone?

**Figure 4.**
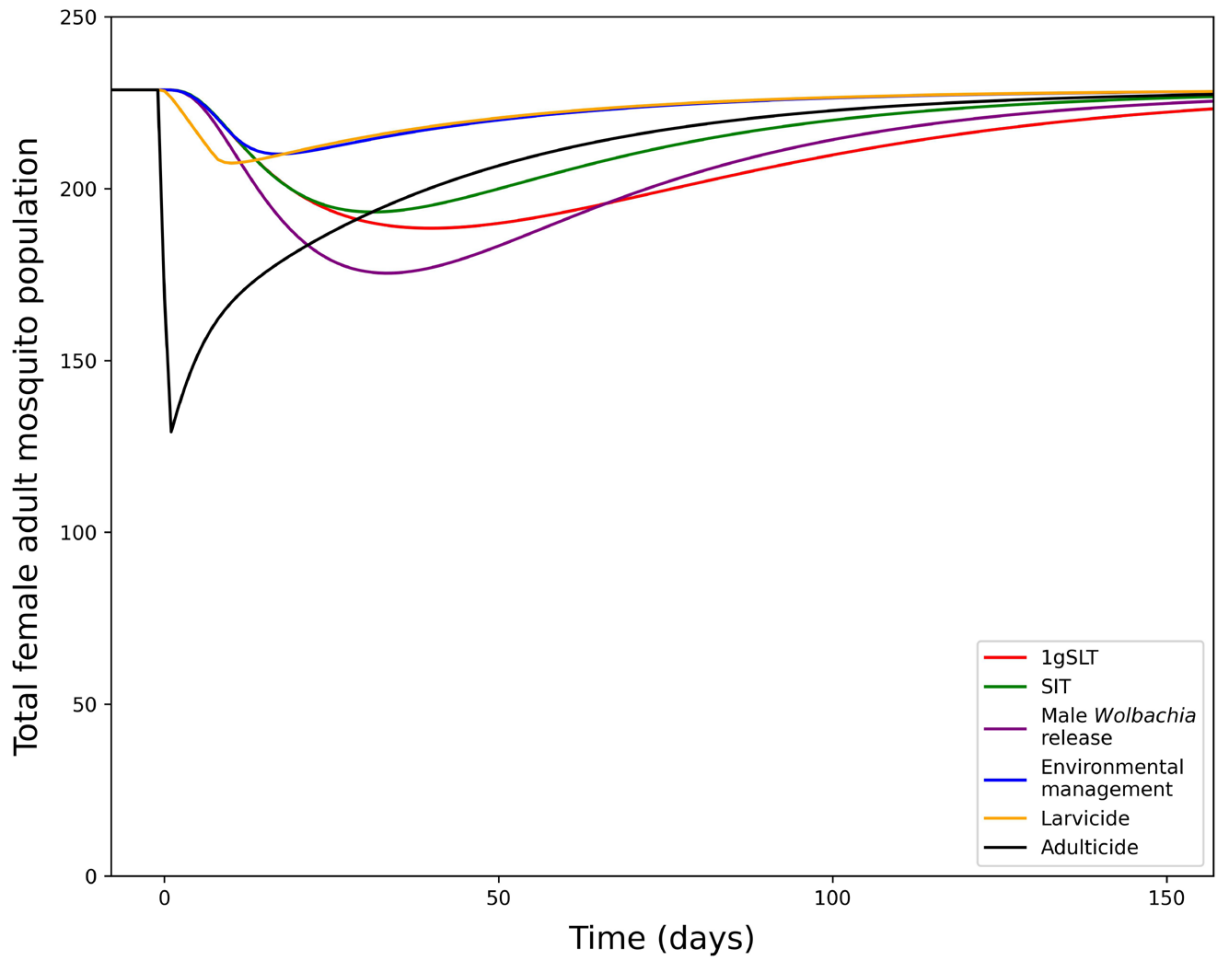
Dynamics of adult mosquito population after a 1-week suppression programme with literature-derived efficacy. Showing the total adult female mosquito population dynamics under 1-week suppression application with different methods.

Development of this model first involved fitting the model to the literature-derived efficacy estimates, which differ for each suppression method. Study design influences measurement of maximum suppression efficacy, time taken to reach maximum suppression, and time taken to recover to pre-suppression levels, which were all considered when fitting the model and making predictions for a standardised single application programme. When comparing a single suppression application with literature-derived efficacy (with SIT assumed to be 50%) adulticide achieves the greatest suppression but rapidly returns to pre-suppression levels ( Figure 4). Other methods take longer to reach peak effectiveness, but also have longer durations of effectiveness, particularly insect release methods. Male *Wolbachia* release is the most effective insect-release method but 1gSLT has a longer-lasting effect. Finally, environmental management and larvicides were estimates to result in the least suppression when applied over a short time period as they typically require long consistent periods of application to reach maximum suppression efficacy. With reductions in adult mosquito population size in the range of 8.18 – 43.51%, from a single application all methods of suppression were predicted to remove the ~1–10% mosquito population exacerbation seen in replacement only programmes.

**Figure 5.**
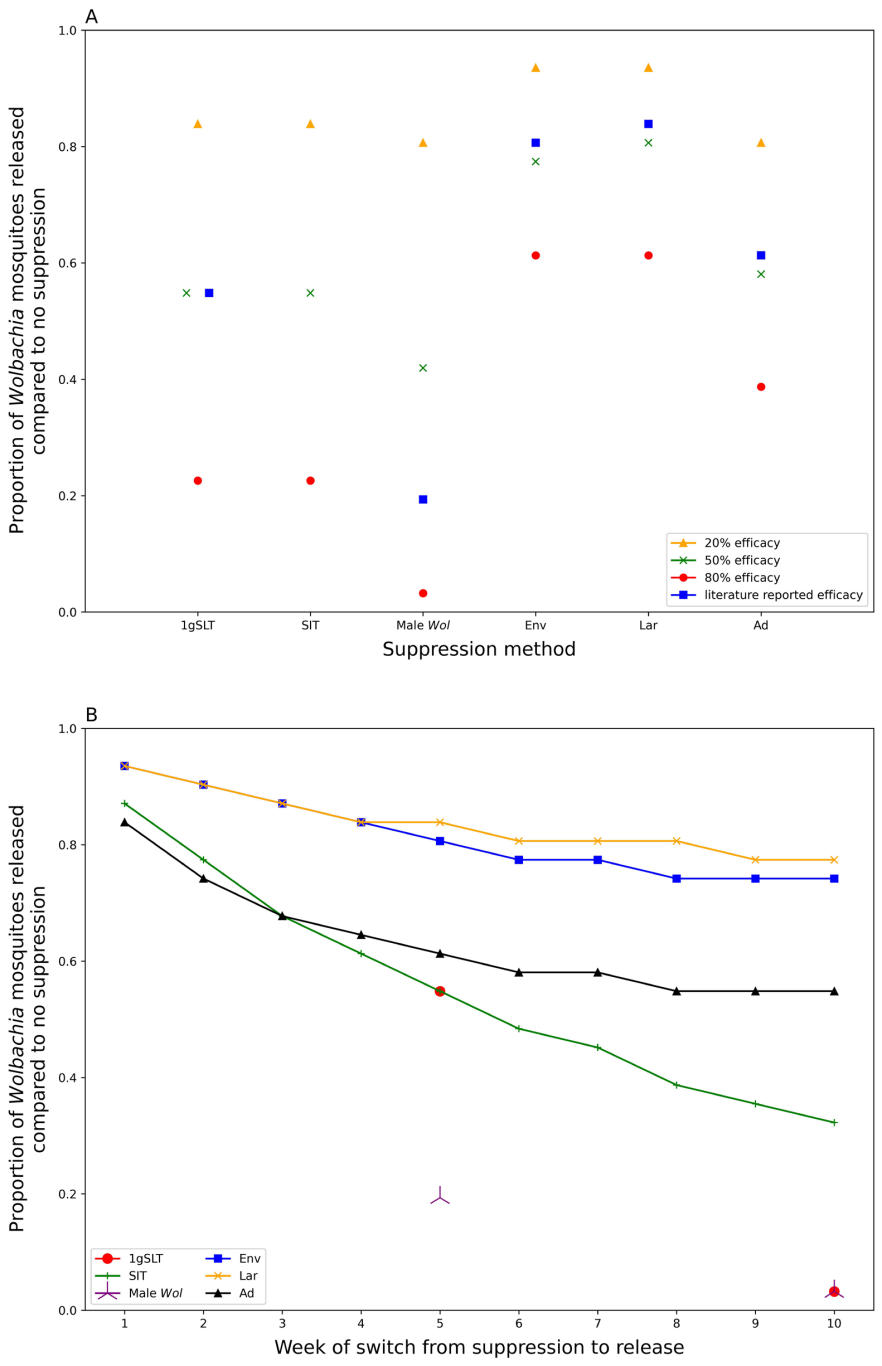
Proportional release ratios for *Wolbachia* replacement programmes post suppression. Replacement with no prior suppression requires a *Wolbachia* release ratio of 0.0775 to achieve target coverage (95%) within 6 months of first release. **A**) shows the proportional reduction in required *Wolbachia* release ratio following a 5-week suppression programme with different methods. **B**) shows how this proportional reduction in required *Wolbachia* release ratio declines with increasing rounds of suppression. All suppression methods use literature-derived efficacy except SIT which uses an assumed 50%. 1gSLT and Male *Wolbachia* release only show two data points because the literature calculated efficacy at 5- and 10-weeks.

Our model predicts that a prior suppression programme of five weekly rounds could reduce the number of *Wolbachia* mosquitoes required to reach target coverage within 6 months by 16-81% depending on suppression method used ( Figure 5A, comparing literature-derived estimates). All insect release-based suppression methods gave greater reductions in required *Wolbachia* mosquitoes than conventional methods. This superiority is maintained even if the peak effectiveness is standardised across different methods of suppression ( Figure 5A), suggesting longer-lasting suppression methods are preferable for hybrid approaches. It may seem counterintuitive to use mosquito killing methods at the same time as *Wolbachia* mosquitoes are being released, but if these mosquito killing methods do not disproportionately affect *Wolbachia* mosquitoes relative to the wildtype (as assumed in our model), suppression will still reduce the overall number of *Wolbachia* mosquitoes required for replacement. Among conventional suppression methods, adulticide outperformed environmental management and larvicide with reductions in required release ratio of 39%, 19% and 16% respectively, considering literature-derived efficacy.

Similar results were found when the number of *Wolbachia* release rounds were reduced (as opposed to reducing the release ratios per round) suggesting programmes could realise this benefit by reducing the number or density of *Wolbachia* releases. Conversely, programmes could choose to release the same number of *Wolbachia* mosquitoes, but now at much higher release ratios which would achieve *Wolbachia* target coverage faster. Insect release suppression methods could be used to decrease the time to target coverage by up to 80%, while conventional methods would only marginally improve speed, or not at all in the case of environmental management ( Table 3).

**Table 3.** Time reductions and potential cost savings enabled by a hybrid approach. * Based on a programme that achieves the TPP minimum target cost of $2.33 per person. Maximum cost savings in the replacement phase assumes suppression has no cost or is an in-kind contribution. ** relative to a baseline programme with nine releases at a relative ratio of 0.09 achieving target coverage (95%) in 215 days.

Method of suppression	Maximum percentage cost reduction	Maximum cost savings in the replacement phase * ($ per person covered)	Percentage reduction in days to achieve target coverage **
**1gSLT**	**18.1 – 50.2**	**$0.42 – 1.17**	**83.4**
**SIT**	**17.4 – 48.3**	**$0.41 – 1.13**	**82.5**
**Male *Wolbachia* release**	**17.4 – 48.3**	**$0.41 – 1.13**	**82.5**
**Environmental ** **management**	**3.8 – 10.7**	**$0.09 – 0.25**	**0.0**
**Larvicide**	**6.4 – 17.8**	**$0.15 – 0.42**	**11.3**
**Adulticide**	**7.7 – 21.4**	**$0.18 – 0.50**	**18.5**

All hybrid programmes had the potential to offer cost savings over replacement alone, depending on the cost of suppression. Mosquito release suppression methods could reduce costs by 18-50% and conventional methods by 4-21% ( Table 3). If the baseline replacement programme can meet the TPP minimum cost target of $2.33 per person, then these percentage reductions would translate to reductions of $0.41 – $1.17 per person for mosquito suppression methods and $0.09 – 0.50 per person for conventional methods. This, however, does not take into account the costs of suppression. A different interpretation of these results would be: if suppression can be achieved for less than these costs, then a hybrid programme will cost less than replacement alone. Even if the costs are equivalent, a hybrid programme may be considered more acceptable from a community perspective due to prevention of the temporary increase in numbers of biting female mosquitoes.

Longer suppression campaigns give diminishing returns when used as part of a hybrid approach ( Figure 5B). The greatest benefits, in terms of reducing *Wolbachia* release requirements, are seen within the first few weeks of suppression with decreasing benefits beyond five weekly rounds of suppression. This effect is more pronounced for insect release suppression methods that see most of their benefits delivered from a one- to three-week suppression programme, while for conventional methods there may still be some benefit in continuing suppression for up to eight weeks ( Figure 5B).

The primary role of these modelling results in developing the PPC was to clarify the likely benefits of the hybrid approach. Namely that a hybrid approach could achieve *Wolbachia* coverage faster, with higher community acceptance and/or at a lower cost. Citing this modelling input the TAG concluded in the PPC that a range of suppression methods can be considered for combination with *Wolbachia* replacement to achieve potential benefits of faster achievement of coverage by *Wolbachia*, higher community and programmatic acceptance and lower cost. The draft PPC states that “trials of a hybrid approach would test the expected benefits of conducting suppression followed by replacement and that modelling thus far suggests that suppression methods involving insect releases will generally reduce the intensity of the *Wolbachia* replacement programme more than other methods”. It was also agreed that modelling would be a useful tool for prioritising intervention combinations for field trials and could be used to directly inform trial design. Finally, the PPC recognises that hybrid approaches may require additional logistical and practical complexities over replacement alone, particularly for mosquito release suppression methods that may require additional regulatory approval. This may mean that rather than hybrid approaches superseding replacement-only approaches, their use may be restricted to areas where replacement-only cannot meet speed, acceptability, and cost goals.

Extension of this modelling work to account for seasonal variations in mosquito population size with the aim of optimising the seasonal timing of replacement-only and hybrid approaches was also requested. Since the original PPC meeting, our model has been expanded to include a typical seasonal profile. Our model assumes mosquito population sizes closely follow variation in precipitation with a 41-day lag and a peak population size ~ 3 times the dry season minimum, consistent with various field observations ^45^ (Supplementary Figure 4, seasonality function sensitivity analysis in Supplementary Table 2).

**Figure 6.**
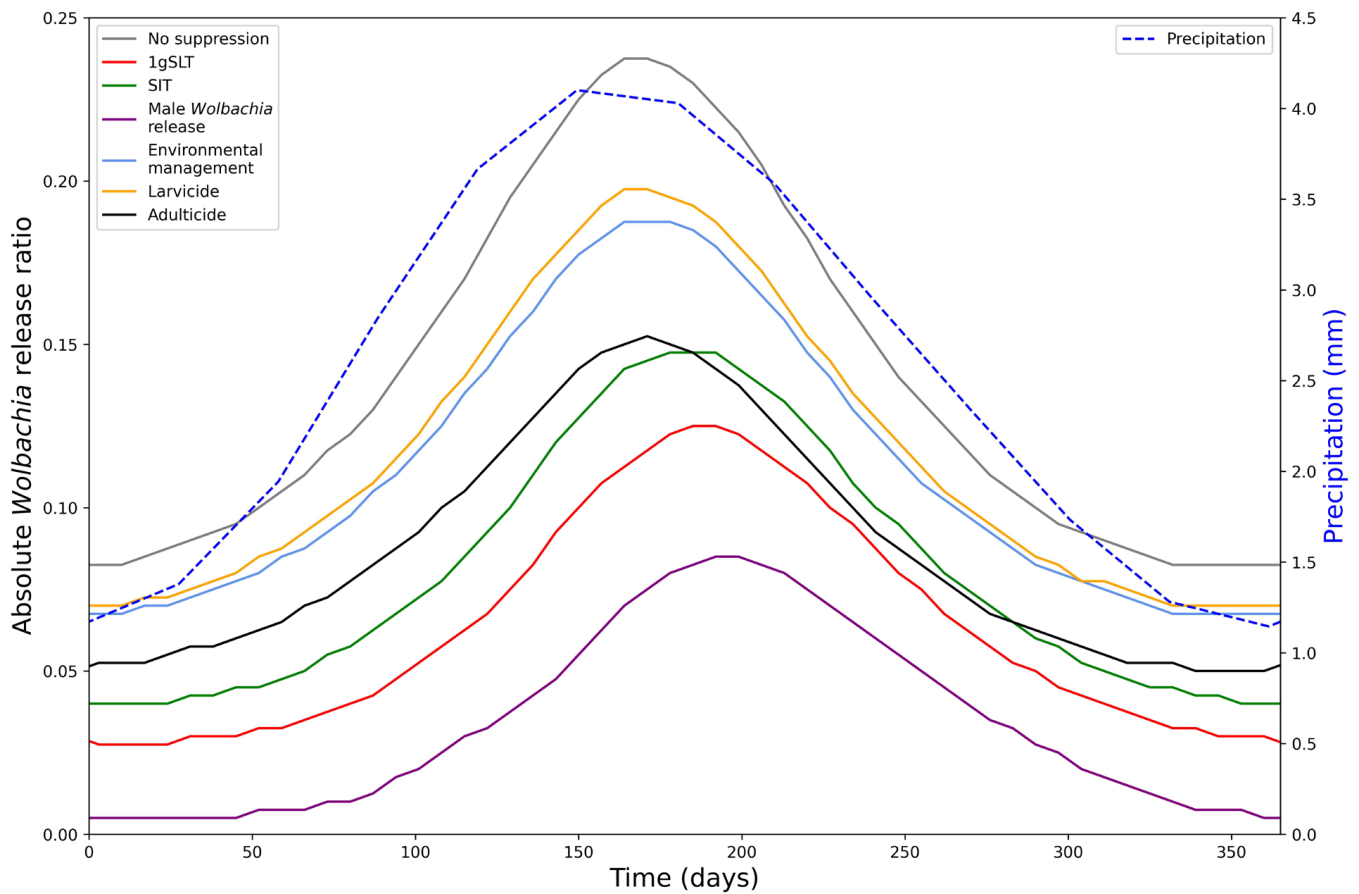
Seasonal variation in hybrid approach effectiveness. Minimum release ratio for a *Wolbachia* replacement programme to reach target coverage (95%) within 6 months of first release after a 5-week suppression programme with different methods. Required release ratio is relative to the annual mean (long-term average) wild type mosquito population. Seasonality in mosquito abundance is simulated by precipitation-dependent larval development.

We predict that the optimal time to begin replacement-only or hybrid programmes is just before the seasonal lowest point in mosquito abundance ( Figure 6). The release ratio required for *Wolbachia* fixation in a replacement only programme mirrors precipitation (and thus wild type mosquito population) dynamics, with a short lag.

When mosquito populations fluctuate throughout the year, the timing of *Wolbachia* replacement has a large effect on the number of Wolbachia mosquitoes that need to be released to reach fixation. Starting the replacement programme at the optimum time can reduce the number of *Wolbachia* mosquitoes by 65.26% compared to the least optimal time. The seasonal scenario also follows the prioritisation of suppression methods observed in the non-seasonal analysis when using a hybrid suppression-then-release approach with male Wolbachia release most effective and larvicide least effective. Furthermore, at the optimal time, in the dry season, the hybrid approach reduces the required *Wolbachia* release ratio by up to 93.94% compared to replacement alone, whereas at the least optimal time, in the wet season, the hybrid approach only reduces the required *Wolbachia* release ratio by up to 64.21%. Finally, because of their delayed effects the insect-release suppression methods (1gSLT, SIT, Male *Wolbachia* release) allow a hybrid strategy to remain effective for longer in the early stages of the wet season, so may be a better choice for areas where the timing of mosquito seasonal cycles is less predictable. 

## Discussion

Mathematical and geostatistical models can make important quantitative and qualitative contributions when developing TPPs and PPCs. Here we show that models can: i) identify important trade-offs, such as the time taken for *Wolbachia* to reach target coverage and the temporary exacerbation in the mosquito population, ii) quantify threshold criteria, such as the $2.33 per person *Wolbachia* replacement cost target, iii) predict characteristics of a product in new areas and at broader scales than it is currently implemented, such as to meet the WHO 25% global burden reduction targets and iv) understand synergies and antagonisms between combinations of products that have not yet been tested, such as a hybrid suppress then replace approach.

Broad community acceptability of *Wolbachia* replacement will clearly be a critical aspect of achieving implementation at the scale envisioned by these TPP and PPC documents. The success of current replacement programmes has been underpinned by extensive community engagement activities ^46, 47^ and other countries (Singapore ^18^ and China ^17^) have chosen to use *Wolbachia* for suppression only, in part, due to concerns over any increases in mosquito abundance. Here we show temporary increases in mosquito abundance can be minimised or avoided entirely by using lower *Wolbachia* release ratios, timing releases to coincide with the dry season, or conducting a prior suppression campaign in a hybrid approach. These steps may, however, involve additional programmatic complexity and likely cost. More work is needed to better understand how mosquito abundance relates to community acceptability in different contexts and how such barriers can be overcome with different release intensities, timings, and hybrid approaches. One alternative use case, considered in the TPP, is to conduct longer lower intensity *Wolbachia* replacement releases to reach an initial target coverage of 50%, after which gaps in coverage are gradually infilled by movement of mosquitoes and auto-dissemination of *Wolbachia*. These lower density releases may have significant cost advantages and could be a more acceptable method of dissemination over broad areas where faster implementation is a lower priority. Such a strategy would, however, take longer, be dependent on patterns of mosquito movement and may be limited by environmental barriers to mosquito spread ^48^. Development of spatial models of mosquito movement and dengue spread could help identify where additional release points may be necessary, target initial release points to high-risk areas and quantify the collateral benefit in disease reduction in neighbouring areas ^49, 50^.

Cost continues to be a barrier to wider adoption of *Wolbachia* replacement when its high costs but long-term benefits are compared to lower cost but short acting suppression methods ^27^. A key strength of our analysis was to link TPP cost targets to conservative estimates of averted costs based on direct medical costs and emergency vector control expenditure over limited timeframes. This was critical to identify geographic differences in cost targets between, but also within countries. Pairing this analysis with high resolution global burden and cost maps identified cost targets that are compatible with wider international goals and equitable across a range of settings ^22^. Work is currently underway to validate our approximation of willingness to pay for *Wolbachia* replacement through surveys targeted to key stakeholders in state and federal governments. The maps and models generated in this work could be adapted for planning national *Wolbachia* replacement campaigns and, in particular, could inform how re-use of release resources, variable pricing models, financing and slower release campaigns could be used to meet the TPP cost targets in even the most challenging countries ^27^. Some of this functionality is already available in the freely available Wolbachia Decision-Support tool ( https://wolbachia-tool.netlify.app/tool#map) which makes use of the outputs of this analysis along with other geospatial layers. 

This analysis predicts that *Wolbachia* replacement releases over 924,557km ^2^ could lead to a 25% reduction in dengue burden globally, averting US$3.05 billion (2.62 – 3.96) worth of medical and outbreak response costs per annum. This may appear ambitious for a minimum product but can be understood in the following context. Although the lowest averted cost/person covered in this area is predicted to be US$2.33, all other release areas could support substantially higher costs ( Figure 3). This means that a *Wolbachia* replacement product would still meet the TPP targets if initial programme costs were higher and if the product has the potential to reduce costs down to the $2.33 target. It is important to note that the TPP only requires that the product is suitable and available everywhere within the target areas, not that it is necessarily implemented in all suitable areas. In practice, commercial considerations, including the need to build capacity, to access funding that is incremental to the current routine control budgets, and to compete and combine with other methods, will limit the rate of uptake and the ultimate scale of deployment achieved.

Hybrid approaches offer one promising option for increasing speed, reducing cost and increasing the acceptability of *Wolbachia* replacement. The models presented here and the wider evidence provided to the PPC support field trials of hybrid approaches as a next logical step. These models can guide the prioritisation of suppression methods, trial sample size calculations and suggest how effectiveness should be measured. We predict that insect-based suppression methods (1gSLT, SIT, Male *Wolbachia* release) will be more effective than conventional suppression tools, but also outline a limited cost window which may be challenging for insect-based suppression methods to achieve. Investment in new infrastructure to conduct insect-based suppression may not be justified for a one-off suppression, but between overlapping resource requirements for suppression and replacement, ongoing use post-replacement (e.g., outbreak control or to achieve dengue elimination) and a continued drive to lower costs of mosquito suppression ^51^, this investment cost may be justified. Intervention developers and countries must ultimately decide how to balance cost and efficacy when considering hybrid approaches. Timing replacement to coincide with the seasonal low point of mosquito abundance is an alternative low-cost hybrid approach and would be a useful addition to trials of hybrid approaches. More generally, our results also suggest that suppression methods that have a longer residual effect are likely to be more beneficial in a hybrid approach. This would suggest some emerging vector control methods, including targeted indoor residual spraying (TIRS) ^52, 53^ and Oxitec’s second-generation Friendly™ mosquito technology ^54^ that allows male survival, would also be strong candidates for a hybrid approach and should also be considered for inclusion in modelling and potentially in hybrid field trials.

These models and the results they generate are not without their limitations and clear communication of these limitations was an important part of their use for the TPP and PPC. Our models of *Wolbachia* replacement do not include any spatial, temporal (beyond seasonal) or stochastic heterogeneities that may mean our model overestimates the speed to achieve fixation and target coverage, particularly in the latter stages (Supplementary Figure 6). We also do not account for a natural wild type egg bank emergence which can dilute *Wolbachia* release ratios, possibly accounting for the low RR values estimated in these analyses. The *Wolbachia* replacement system targets *Ae. aegypti*, which is the major vector of dengue, but *Ae. albopictus* can be locally important and the need for a Wolbachia replacement product to control both species is not considered in this modelling. *Ae. albopictus* and other mosquito species are assumed to be controlled by routine vector control programmes which would be ongoing in parallel with any *Wolbachia Ae. aegypti* replacement or hybrid control programmes. There remain large gaps globally in data on the cost of dengue treatment and prevention and no comprehensive cost estimates for Zika, chikungunya or yellow fever, all of which *Wolbachia* will provide some efficacy against. Cost estimates are therefore generally conservative and should not replace primary data on willingness to pay or more detailed cost-benefit analyses when considering programmes in any one given country. We also recognise that only one modelling group was included for this TPP and PPC and that inclusion of multiple modelling teams can help better represent the structural uncertainty of models and their interpretation when deciding between policy options ^55^. Fitting each suppression method to literature reported effectiveness estimates was challenging due to incomparable ways in which suppression was implemented and evaluated, and therefore the modelling outputs for each method may not be representative. In particular, our chosen source of evidence for adulticide suppression reported a 94% followed by a return to pre-suppression population within 7 days ^42^, a rate of rebound that our model was unable to replicate from newly emerging adult mosquitoes alone, thus we had to assume that the 94% effectiveness was only achieved in a fraction of the overall mosquito population. Moreover, this efficacy is higher than generally expected for outdoor space-spraying in urban environments in practice ^56^. Our hybrid approach results also assume that the suppression method acts independently of any other forms of vector control already in use in the area. Suppression will likely be reduced if the method of suppression (or similar methods that use the same modes of action or insecticides) is already routinely used.

In this paper we show the value dedicated modelling research can add to the development of TPPs and PPCs. By making the data, code and fitted models freely available to accompany the TPP for *Wolbachia* replacement and PPC for the hybrid approach, product developers are able to continue to use and adapt them to steer the development of a range of *Wolbachia* products to meet the rising challenge of global dengue control.

## Ethics

No ethical approval was necessary as all data used was publicly available.

## Data Availability

GitHub. Using models and maps to inform Target Product Profiles and Preferred Product Characteristics: the example of Wolbachia replacement. DOI: https://github.com/katietiley/Wolbachia_TPP_PPC.git This project contains the following data: All data and code used for each of the models used in this analysis and the predictions made by these models Supplementary file 1 and all supplementary figures referred to in the main text Data is available under the MIT Licence.
