# Supplementary material for: Using models and maps to inform Target Product Profiles and Preferred Product Characteristics: the example of Wolbachia replacement
Source: Gates Open Res. 2024 Oct 31;7:68. Originally published 2023 May 12. [Version 3] doi: 10.12688/gatesopenres.14300.3 (PMC11549085; doi:10.12688/gatesopenres.14300.3)
Supplement: Peer review history

# Reviewer report: Reviewer response for version 3

**DOI:** 10.21956/gatesopenres.17692.r38381

**Published:** 2024-11-08

**Authors:** Perran Ross

**Affiliations:** 1 The University of Melbourne, Melbourne, Victoria, Australia

I thank the authors for addressing comments from myself and the other reviewers in Version 3. While the changes in Version 2 were relatively minor and did not satisfactorily address my key concerns, the authors have now expanded their discussion to highlight several assumptions and limitations. While I still disagree with the authors on some fundamental points (e.g. the value of direct comparisons between suppression techniques), my main concerns have been addressed (e.g. statements that hybrid suppress then replace releases constitute cost savings) and I would be happy to approve this version.

Is the work clearly and accurately presented and does it cite the current literature?

Partly

If applicable, is the statistical analysis and its interpretation appropriate?

No

Are all the source data underlying the results available to ensure full reproducibility?

Yes

Is the study design appropriate and is the work technically sound?

No

Are the conclusions drawn adequately supported by the results?

No

Are sufficient details of methods and analysis provided to allow replication by others?

Yes

Reviewer Expertise:

Wolbachia-based mosquito control

I confirm that I have read this submission and believe that I have an appropriate level of expertise to confirm that it is of an acceptable scientific standard.

# Reviewer report: Reviewer response for version 2

**DOI:** 10.21956/gatesopenres.17127.r37731

**Published:** 2024-09-30

**Authors:** Asha Mary Abraham

**Affiliations:** 1 Christian Medical College, Vellore, India

This paper describes the application of entomological and economic modelling for *Wolbachia* replacement in *Aedes egypti* mosquitoes as a method to decrease the burden of diseases spread by these mosquitoes (mainly dengue, Zika, yellow fever and chikungunya) in the absence of widely available vaccines (apart from that for yellow fever) and antiviral therapy. While the traditional methods of vector control (environmental management and application of larvicides and adulticides) do not control the mosquitoes effectively over the long term, novel technologies are currently under development of which *Wolbachia spp* have been used to replace, reduce and suppress these mosquitoes. Other technologies include first generation self-limiting (1gSLT) and sterile insect (SIT) technologies.

 Aligning with The World Health Organisation’s (WHO) goal to reduce dengue incidence by 25 % by 2030, the authors aimed to describe how these models can aid the development of Target Product Profiles (TPPs) for *Wolbachia* replacement and Preferred Product Characteristics (PPCs) documents for hybrid mosquito suppression followed by *Wolbachia* replacement. 

 The entomological model used data from the Yogyakarta RCT on *Wolbachia*-infected mosquito release, where the delivery mechanism was egg releases in release cups. The detailed statistical formulae used along with the description of the parameters involved are all given clearly for suppression of *Wolbachia*-infected mosquitoes as well as for initial intervention followed by *Wolbachia*-infected mosquito egg release. These initial interventions include release of *Wolbachia*-infected, 1gSLT- and SIT-derived adult males, as well as environmental management using larvicides and adulticides. The seasonality profile with distinct wet and dry seasons annually was produced with data from Rio de Janeiro, and this function was included in the model with respect to precipitation as well as temperature.

 Various release scenarios were analysed at different suppression efficacies, week of switch from suppression to *Wolbachia* release with increments of release ratios needed to reach target coverage within 6 months of first release. The seasonality model was run for a year (the first 6 months of an 18-month run being discarded) with 5 weeks of suppression followed by 5 rounds of *Wolbachia* replacement release with minimum release rates simulated to reach target coverage.

 The economic model was designed to give high spatial resolution estimates of costs of dengue that would be averted by *Wolbachia *replacement, comprising direct medical costs of treating dengue and outbreak vector control costs. 

 The authors estimate that the cost to reduce the global burden of dengue by 25% by the year 2030 is between US$ 7.63 to US$ 0.24 per person protected  or less.

 The specific clarifications I have relate to:

 1. The economic model: the comment that cost savings from the hybrid programmes offer savings over replacement alone programmes is misleading since the cost of the additional suppressive methods  has to be included in addition to replacement.

 2. For large developing countries like 

 India, even the lowest cost per person protected in the predicted range works out to an enormous sum across the large population. It may actually prove more cost-effective to evaluate newer vaccines for dengue (with more specific neutralising antibody production and hence, less ADE activity) as has currently begun.

 3. Regarding the environmental model, can weather data from Rio de Janeiro be applicable to other countries in the tropics?

Is the work clearly and accurately presented and does it cite the current literature?

Yes

If applicable, is the statistical analysis and its interpretation appropriate?

I cannot comment. A qualified statistician is required.

Are all the source data underlying the results available to ensure full reproducibility?

Yes

Is the study design appropriate and is the work technically sound?

Partly

Are the conclusions drawn adequately supported by the results?

Partly

Are sufficient details of methods and analysis provided to allow replication by others?

Partly

Reviewer Expertise:

Dengue and other arbovirus diagnostics and research including cell culture, serology, molecular assays.

I confirm that I have read this submission and believe that I have an appropriate level of expertise to confirm that it is of an acceptable scientific standard, however I have significant reservations, as outlined above.

# Response

**Published:** 2024-10-14

**Authors:** Katie Tiley

Thank you for reviewing this manuscript, we have addressed each of your comments.

***1.*** *The economic model: the comment that cost savings from the hybrid programmes offer savings over replacement alone programmes is misleading since the cost of the additional suppressive methods has to be included in addition to replacement.*

**Response:** In response to another reviewer’s suggestion, the manuscript has now been edited throughout to remove statements indicating that a combined suppress and then release programme presents cost savings.

***2.*** *For large developing countries like India, even the lowest cost per person protected in the predicted range works out to an enormous sum across the large population. It may actually prove more cost-effective to evaluate newer vaccines for dengue (with more specific neutralising antibody production and hence, less ADE activity) as has currently begun.*

**Response:** We agree that total programme costs can appear large when scaled up over populous countries. For clarity, our approach does not project scaling up *Wolbachia *to cover every area in India or elsewhere, but instead targets *Wolbachia* to a subset of high burden areas, which can help to decrease costs. We do acknowledge, however, that other methods of dengue control will likely be important in reaching the WHO 2030 goals, including vaccines, particularly in areas where *Wolbachia* implementation may be challenging or less cost effective. We have edited the discussion to clarify this:

 “In practice, commercial considerations…will limit the rate of uptake and the ultimate scale of deployment achieved. In areas where *Wolbachia* implementation may be challenging or less cost effective, other methods of dengue control, including vaccines, will likely be important in reaching the WHO 2030 goals.”

***3.*** *Regarding the environmental model, can weather data from Rio de Janeiro be applicable to other countries in the tropics?*

**Response: **While the economic modelling components of our analysis were geographically explicit, the mosquito population dynamics model was intended to represent a more generalisable dengue-endemic setting with distinct wet and dry seasons that drive mosquito population dynamics. Rio de Janeiro has a typical meteorological profile of a dengue-endemic region with distinct wet and dry seasons. Basing our generic model on this typical seasonal profile allowed us to explore the effects of distinct seasonal changes on *Wolbachia* release ratio and other hybrid scenarios. The results of this analysis are now found in Supplementary Figure 7.

 We hope you now find this manuscript acceptable for publication.

# Reviewer report: Reviewer response for version 2

**DOI:** 10.21956/gatesopenres.17127.r37727

**Published:** 2024-09-16

**Authors:** Gabriela Paz-Bailey

**Affiliations:** 1 Centers for Disease Control and Prevention, San Juan, Puerto Rico, USA

The paper reports on a mathematical model used to inform the WHO Target Product Profile (TPP) development for *Wolbachia* replacement. It contributes to the literature by discussing aspects of the TPP that influence *Wolbachia *implementation, using dengue cost data as a proxy for willingness to pay, and highlighting areas with the greatest benefit based on disease burden. However, there are several areas where the paper could be improved:

  

1. The components of the TPP need to be explained in the background section. Some elements are mentioned later in the paper without sufficient context. Adding a table with the TPP elements and their descriptions would be helpful.
2. Several parameters and assumptions in the model conflict with current replacement program practices:

a. Most programs release adult mosquitoes daily, rather than releasing eggs every two weeks. This discrepancy likely impacts costs and time to introgression.

 b. Costs decrease when intervening in larger areas with high population density. Dividing endemic areas into 5x5 km pixels to prioritize regions seems unrealistic. For the model to be useful, it should reflect how countries are likely to implement the strategy, prioritizing cities based on population density and dengue incidence. What is the point of having a few pixels in one country and a few in another on the priority list if implementing the strategy based on these priorities is not feasible?

  

1. The focus on the combination of suppression followed by replacement is disconnected from practical realities. There are only two places in the world with *Wolbachia *suppression program (Singapore and China), and these do not represent the capabilities of endemic countries. This topic is prominently featured in the background, introduction, results, and discussion. I recommend moving it to the supplemental information and including a sentence in the results directing readers to it. Presenting it so prominently could distract from the main message and might influence policy and implementation discussions inappropriately. The main challenges with mosquito control include insecticide resistance, the limited impact of ULV spraying, and the difficulty of reaching cryptic breeding sites with larvicides. Mosquito control is largely ineffective and suggesting it as a viable strategy to accelerate replacement programs is dangerous. Effectiveness of SIT and *Wolbachia* methods have not been demonstrated outside a few small trials or in high-resource settings like Singapore and China. Wolbachia suppression is also potentially more expensive than the six-month replacement releases. Claiming that it may reduce costs without including the costs of various suppression methods is misleading. Table 3 should be eliminated.
2. Results:
  1. The results would benefit from examples to aid interpretation. For instance, figures 2c and 2d, and throughout the results section.
  2. Figure 1: The reduction in mosquito population has not been demonstrated in field trials. It would be important to show whether the model results can be corroborated by empirical data.
  3.  Figure 2 highlights the challenges of the pixel approach. Covering only 12.5% of the burden means that smaller areas spread further apart would need to be covered, which may not be feasible with an intervention as logistically demanding as *Wolbachia* replacement. This should be noted somewhere in the paper.
  4. Page 10, third paragraph: Explain what is meant by "exacerbation issues."
  5. Table 2: The implications of the cost per person covered could be better explained in the results. Providing an example of a city with a certain population and comparing the costs of protection in areas with high versus low willingness to pay would be useful. The significance of the cost changes from 12.5% to 25% reduction is unclear.
3. Discussion: a. Page 17, first paragraph: In countries where Wolbachia has been implemented, releases stop when *Wolbachia *introgression reaches 50-60%, allowing it to continue to naturally progress to a higher prevalence. Proposing this as an alternative to current practice is wrong as it is already how it is done.

Is the work clearly and accurately presented and does it cite the current literature?

Yes

If applicable, is the statistical analysis and its interpretation appropriate?

Partly

Are all the source data underlying the results available to ensure full reproducibility?

No source data required

Is the study design appropriate and is the work technically sound?

Yes

Are the conclusions drawn adequately supported by the results?

Partly

Are sufficient details of methods and analysis provided to allow replication by others?

Yes

Reviewer Expertise:

Epidemiology

I confirm that I have read this submission and believe that I have an appropriate level of expertise to confirm that it is of an acceptable scientific standard, however I have significant reservations, as outlined above.

# Response

**Published:** 2024-10-14

**Authors:** Katie Tiley

*The paper reports on a mathematical model used to inform the WHO Target Product Profile (TPP) development for Wolbachia replacement. It contributes to the literature by discussing aspects of the TPP that influence Wolbachia implementation, using dengue cost data as a proxy for willingness to pay, and highlighting areas with the greatest benefit based on disease burden. However, there are several areas where the paper could be improved:*

***1.*** *The components of the TPP need to be explained in the background section. Some elements are mentioned later in the paper without sufficient context. Adding a table with the TPP elements and their descriptions would be helpful.*

**1. **Thank you for reviewing this manuscript. We agree that more context on the TPP would be helpful. This is boosted by the final approved version of the TPP now being publicly available which we now reference and should provide additional detail if readers are interested. We have expanded the relevant section of the introduction to summarise the main features of the TPP:

 “In early 2022 the WHO convened a Technical Advisory Group (TAG) to develop a draft TPP for Wolbachia replacement and a draft PPC for a hybrid mosquito suppression then Wolbachia replacement strategy. The TPP for Wolbachia replacement began with the development of a “use case characterisation”, following which specific TPP criteria were established under the categories of product performance, product characteristics, production and delivery and intellectual property. For each of these, a minimum and preferred target was established with the former intended to inform a go / no go product development decision point. A combination of different types of evidence from the field, laboratory and modelling studies were used to inform these targets, with the modelling work focused on release and cost-related characteristics. The final WHO TPP was published February 2022 (World Health Organisation, 2022). TAG members decided that a core premise of the TPP and PPC was that they should closely align with the WHO’s strategy and goals to control dengue globally. As such the WHO’s goal to reduce dengue incidence by 25% by 2030 (2010 – 2020 baseline24) provided a basis to understand the scale and range of settings in which these TPPs, PPCs and the products they ultimately produce are relevant.”

*Several parameters and assumptions in the model conflict with current replacement program practices:*

***1a.*** *Most programs release adult mosquitoes daily, rather than releasing eggs every two weeks. This discrepancy likely impacts costs and time to introgression.*

**1a. **We acknowledge that the form and frequency of releases may have changed since the time this decision was made. Our focus on egg releases every two weeks was aligned with the major field evidence available at the time which was the Yogyakarta RCT (Utarini et al., 2021) which also informed other aspects of the TPP. Therefore, this choice was made to improve consistency with the available field data and consistency across different elements of the TPP. We have edited the text in the methods to emphasise this:

 “An entomological Wolbachia replacement model was formulated and calibrated to the main evidence available at the time, namely the Yogyakarta RCT which comprised nine to 14 egg releases in release cups every two weeks. This approach was also chosen to maximize consistency with other elements of the TPP.”

 We have also added an additional limitation to the discussion:

 “While we simulate egg releases once every two weeks, we acknowledge that adult releases at weekly timescales may be possible and could accelerate the time to fixation.”

***1b.*** *Costs decrease when intervening in larger areas with high population density. Dividing endemic areas into 5x5 km pixels to prioritize regions seems unrealistic. For the model to be useful, it should reflect how countries are likely to implement the strategy, prioritizing cities based on population density and dengue incidence. What is the point of having a few pixels in one country and a few in another on the priority list if implementing the strategy based on these priorities is not feasible?*

**1b.** We agree with the review that treating pixels in isolation is likely not realistic. The same point was raised by review #1 in their initial review (see 1.2.1b for comment and response). In this response we clarified that occurrence of isolated 5x5km pixels is very rare in our analysis. We summaries the treated area estimates for each 2 ^nd^ administrative area and provide this in the supplementary information.

 Original response: “The administrative-unit summary provided in the supplement shows that 85.5% of 2nd administrative units (admin2s) have predicted release areas at or above 10km2. Among the remaining admin2s with smaller release areas all of them are in countries that also contain admin2s with larger (>10km2) release areas and 66% of smaller release admin2s occur in Vietnam, Colombia, Thailand, Brazil and Mexico which would all require large release areas across their respective countries. These smaller areas could thus be considered an extension of programmes in nearby areas. Introducing restrictions on contiguity or release area will, therefore, only affect a very small number of areas and have minimal impact on the overall findings.”

 In this response we also acknowledge the gap between our predicted treated areas map and likely implementation area:

 “We recognize and agree that many additional considerations will go into which areas will ultimately be targeted for Wolbachia implementation and the resulting map is not intended to be prescriptive, but rather give an indication of the kinds of areas that would be most sensible to priorities from a net cost efficiency perspective. Even if the decision to implement occurs at administrative unit-level, not everywhere within an administrative unit will see Wolbachia releases and the pixel-level maps can be useful for informing within- administrative unit targeting subject to a set of criteria that should be locally determined. It should also be emphasized that the TPP is, by definition, for a hypothetical Wolbachia replacement product, not the current most widely-used wMel programme. This means that such considerations may differ in their importance and, as such, are beyond the scope of the TPP and the accompanying modelling analysis.”

***2.*** *The focus on the combination of suppression followed by replacement is disconnected from practical realities. There are only two places in the world with Wolbachia suppression program (Singapore and China), and these do not represent the capabilities of endemic countries. This topic is prominently featured in the background, introduction, results, and discussion. I recommend moving it to the supplemental information and including a sentence in the results directing readers to it. Presenting it so prominently could distract from the main message and might influence policy and implementation discussions inappropriately. The main challenges with mosquito control include insecticide resistance, the limited impact of ULV spraying, and the difficulty of reaching cryptic breeding sites with larvicides. Mosquito control is largely ineffective and suggesting it as a viable strategy to accelerate replacement programs is dangerous. Effectiveness of SIT and Wolbachia methods have not been demonstrated outside a few small trials or in high-resource settings like Singapore and China. Wolbachia suppression is also potentially more expensive than the six-month replacement releases. Claiming that it may reduce costs without including the costs of various suppression methods is* *misleading. Table 3 should be eliminated.*

**2.** The suppression then replacement strategies were developed for the PPC. We explicitly clarify in the Introduction that:

 “PPCs identify broader areas of unmet need and aim to stimulate new products or product combinations that can address these needs.”

 This work, therefore, is not intended to represent current practical realities, but rather understand under what circumstances such new strategy may be beneficial. As well as having a clear theoretical basis, this intervention combination has been suggested by multiple previous modelling studies (Hu et al., 2021; Qu et al., 2018; Yakob et al., 2008) and is therefore a plausible potentially viable strategy that should be tested. We believe that the results presented in this paper are not particularly favorable for suggesting the hybrid approach over conventional replacement and list extensive barriers that will need to be overcome for it to become a viable alternative. We don’t believe removing or relegating this work to the supplementary information would remove any perceived danger as implementers could still ask about these strategies based on work in prior publications. While we agree that the results text for the hybrid strategies is longer than for conventional replacement, this reflects the additional model development and complexity more than any perceived prominence or prioritization between these options.

 We do, however, agree that we can do more to differentiate the work done for the TPP and PPC and as a result, we have made a number of modifications to sections to clarify that hybrid strategies are not currently used and the caveats that would need to be addressed for it to become a practical reality.

 In the introduction:

 “While not currently practiced, in theory, combining a prior programme of mosquito suppression followed by Wolbachia population replacement could offer community acceptance or dengue incidence reduction advantages.”

 In the discussion:

 “Hybrid approaches offer one unproven but potential option for increasing speed or acceptability of Wolbachia replacement.”

 And

 “The implementation of any suppression method should be accompanied by mosquito surveillance that confirms that the levels of suppression achieved are above and beyond what would have normally been achieved by the programme.”

 Furthermore, Figure 6 showing a seasonal analysis of hybrid suppress then release strategies has been moved to the Supplementary Materials.

 Following the comments of reviewer #1 all reference to the cost savings of hybrid suppress then release programmes have been removed and we substantially modified Table 3 and the section of text where it is discussed:

 “By reducing the number of Wolbachia mosquitoes released and duration of the replacement phase of the hybrid strategy, the costs of this phase could be reduced. If these levels of suppression can be achieved at a lower cost than the savings of the replacement phase, then a hybrid strategy could reduce costs overall. Here we use models to estimate these maximum costs for suppression, beyond which a replacement-only programme would be preferable. If the baseline replacement programme can meet the TPP minimum cost of $2.33 per person, then suppression would need to cost not more than $0.41 – $1.17 per person for mosquito-release suppression methods and $0.09 – 0.50 per person for conventional methods (Table 3).”

*Results:*

***3a.*** *The results would benefit from examples to aid interpretation. For instance, figures 2c and 2d, and throughout the results section.*

**3a. **While we appreciate the suggestion, including these is challenging due to the fact that all well-known example cities are included in all of the targeting scenarios and our desire to keep the focus at a global level (see response 4e). We have slightly modified parts fo the results where figure 3 is described:

 “This corresponds to 34.7% of the urban (> 300 people per km2) area at risk and all major dengue-endemic cities, and just 1.7% of the total area at risk of dengue.”

 And

 “A full list of cities and 2nd administrative units included under each targeting scenario, the costs each will support and additional contextual information (population, density, etc) is included in the following repository: https://github.com/katietiley/Wolbachia_TPP_PPC.git.“

***3b.*** *Figure 1: The reduction in mosquito population has not been demonstrated in field trials. It would be important to show whether the model results can be corroborated by empirical data.*

**3b. **While we agree that measuring pre- and post- *Wolbachia* replacement mosquito population size in the field is important, we are currently not aware of any datasets where this has been robustly measured and reported. We believe this may be challenging to practically measure given the heterogeneity over time and space of mosquito populations and the variable measurement accuracy of different traps for estimating true mosquito abundance.

 The modest (~ 30-35%) declines in mosquito abundance that we estimate are an implicit consequence of reduced *Wolbachia* mosquito fitness, a trait that is well documented in laboratory studies and a common assumption of many modelling studies (e.g., Joubert et al., 2016; Ross & Hoffmann, 2022). In comparison with these, the fitness cost we selected was a conservative estimate which, consequently, means our estimates of mosquito abundance reduction post- *Wolbachia* release are more likely to be an over-estimate. We have edited the discussion to acknowledge this:

 “The fitness cost of *Wolbachia* infection selected in these models was a conservative estimate and, consequently, the decline of total mosquito population equilibrium from pre- to post- *Wolbachia* release (Figure 1A,B) is likely to be an over-estimation.”

*Figure 2 highlights the challenges of the pixel approach. Covering only 12.5% of the burden means that smaller areas spread further apart would need to be covered, which may not be feasible with an intervention as logistically demanding as Wolbachia replacement. This should be noted somewhere in the paper*

 In the discussion we mention:

 “It is important to clarify that these maps should not be used prescriptively, but rather give an indication of the kinds of areas that are likely to be most cost efficient to target, with the final decision on which areas are targeted for release subject to additional operational, entomological, financial and political considerations.”

***3c.*** *Page 10, third paragraph: Explain what is meant by "exacerbation issues”.*

**3c. **This has been amended:

 “Higher RRs and higher release round numbers also lead to disproportionately undesirable temporary exacerbation of the mosquito population, particularly at higher values (Figure 1A,B,D).”

***3d.*** *Table 2: The implications of the cost per person covered could be better explained in the results. Providing an example of a city with a certain population and comparing the costs of protection in areas with high versus low willingness to pay would be useful. The significance of the cost changes from 12.5% to 25% reduction is unclear.*

**3d. **We appreciate the review’s implementer focussed perspective, but this was not the intended purpose of this model, or these results and we believe that discussing such exampled explicitly may lead to misinterpretation of this work. The cost targets presented in Table 2 are global targets that a Wolbachia replacement solution would need to meet to be compatible with the TPP. While it is true that for any one individual city, these targets could be higher (indicating higher willingness to pay), development of a Wolbachia replacement product at this cost point would not then achieve the TPP target of being affordable in enough areas to meet either the 12.5% or 25% global burden reduction targets. We therefore believe focussing the discussion of these results at the global level is a more appropriate summary of the model and its results.

*Discussion:*

***4a.*** *Page 17, first paragraph: In countries where Wolbachia has been implemented, releases stop when Wolbachia introgression reaches 50-60%, allowing it to continue to naturally progress to a higher prevalence. Proposing this as an alternative to current practice is wrong as it is already how it is done.*

**4a. **The aim of this sentence is to indicate an alternative use case where Wolbachia is released in a checkerboard spatial pattern where the current standard release programme is completed in 50-60% of areas and, over time, mosquito movement allows Wolbachia to spread to non-release areas to gradually build up to 90% of areas covered. This is distinct from prevalence within a single area, where we agree that the current practice is to release Wolbachia mosquitoes until population prevalence is ~50-60% then relying on cytoplasmic incompatibility to further increase prevalence to 90% after releases stop.

 Thank you for highlighting how the previous phrasing may be misleading. We have now edited to clarify how the alternative TPP use case differs from current practice and hope this improves interpretation:

 “One alternative use case, considered in the TPP, is to conduct lower intensity releases with larger distances between release sites. This would result in only around 50% of areas initially having a Wolbachia prevalence of over 90%, however over time mosquito diffusion would spread Wolbachia to all remaining areas to ensure all target areas had a Wolbachia prevalence over 90%.”

References

 Hu, L., Yang, C., Hui, Y., & Yu, J. (2021). Mosquito Control Based on Pesticides and Endosymbiotic Bacterium Wolbachia. Bulletin of Mathematical Biology, 83(5). https://doi.org/10.1007/s11538-021-00881-9

 Joubert, D. A., Walker, T., Carrington, L. B., De Bruyne, J. T., Kien, D. H. T., Hoang, N. L. T., Chau, N. V. V., Iturbe-Ormaetxe, I., Simmons, C. P., & O’Neill, S. L. (2016). Establishment of a Wolbachia Superinfection in Aedes aegypti Mosquitoes as a Potential Approach for Future Resistance Management. PLoS Pathogens, 12(2). https://doi.org/10.1371/journal.ppat.1005434

 Qu, Z., Xue, L., & Hyman, J. M. (2018). Modeling the transmission of wolbachia in mosquitoes for controlling mosquito-borne diseases*. SIAM Journal on Applied Mathematics, 78(2), 826–852. https://doi.org/10.1137/17M1130800

 Ross, P. A., & Hoffmann, A. A. (2022). Fitness costs of Wolbachia shift in locally-adapted Aedes aegypti mosquitoes. Environmental Microbiology, 24(12), 5749–5759. https://doi.org/10.1111/1462-2920.16235

 Utarini, A., Indriani, C., Ahmad, R. A., Tantowijoyo, W., Arguni, E., Ansari, M. R., Supriyati, E., Wardana, D. S., Meitika, Y., Ernesia, I., Nurhayati, I., Prabowo, E., Andari, B., Green, B. R., Hodgson, L., Cutcher, Z., Rancès, E., Ryan, P. A., O’Neill, S. L., … Simmons, C. P. (2021). Efficacy of Wolbachia-Infected Mosquito Deployments for the Control of Dengue. New England Journal of Medicine, 384(23), 2177–2186. https://doi.org/10.1056/nejmoa2030243

 World Health Organisation. (2022). TPP Wolbachia Aedes aegypti population replacement. https://cdn.who.int/media/docs/default-source/ntds/vector-ecology-mangement/tpp-wolbachia-infected-aedes-aegypti-population-replacement-intervention.pdf?sfvrsn=5296c1dc_3

 Yakob, L., Alphey, L., & Bonsall, M. B. (2008). Aedes aegypti control: The concomitant role of competition, space and transgenic technologies. Journal of Applied Ecology, 45(4), 1258–1265. https://doi.org/10.1111/j.1365-2664.2008.01498.x

# Reviewer report: Reviewer response for version 2

**DOI:** 10.21956/gatesopenres.17127.r36946

**Published:** 2024-07-18

**Authors:** Scott O'neill

**Affiliations:** 1 World Mosquito Program, Monash University, Clayton, Victoria, Australia

In general, the manuscript has been improved and is appropriately more qualified in this second version. I still have one problem though which I think is very important as I feel that as it currently stands the paper is quite misleading and this relates to the notion that hybrid release programs would be more cost effective than replacement alone.

 My specific comments on this point are as follows:

 In the introduction it is stated:

 “Specifically, a programme of suppression followed by replacement has the potential to increase the likelihood of successful *Wolbachia* establishment and reduce the cost of, and risk of mosquito exacerbation associated with, achieving establishment ^22^.”

 The paper does not provide any evidence that costs could be reduced by combining suppression and replacement – only if suppression is considered as being free, which in no context is that the case. This statement relating to cost should be removed. I have no problems with the other attributes of the potential benefits.

 In the hybrid “suppress then replace” section it is stated that:

 “This, however, does not take into account the costs of suppression. A different interpretation of these results would be: if suppression can be achieved for less than these costs, then a hybrid programme will cost less than replacement alone. This might refer to specific circumstances when suppression might be achieved at negligible cost, for example, if it’s already part of a vector control programme. These are ideal situations where the resources for suppression are already in place, however, in most cases the potential savings achieved by a hybrid suppress then replace programme won’t justify the additional cost of implementing a novel suppression programme.”

 The notion that if suppression activities are already part of an existing vector control programme that they can be considered to have no cost is nonsensical. Of course they have cost to the government and the economics needs to support total costs of a dengue control program, not just a section of the cost. Finally, the authors are proposing that insect release technologies to undertake suppression would be most effective from a suppression perspective but there are no examples of these costs being less than the cost savings projected from a shortened replacement program, and indeed the evidence available from Singapore indicates the opposite. Thus the conclusions are unrealistically optimistic for the cost-effectiveness of a hybrid program and need to be more qualified.

 The same criticism relating to cost applies to this sentence in the discussion:

 “Hybrid approaches offer one promising option for increasing speed, reducing cost and increasing the acceptability of *Wolbachia* replacement. “ 

 I think there is no justification for the reducing cost element of this statement. There may be a use case though and that is in contexts where mosquito populations are extremely large as occurs in some geographies, such as Kiribati for example.

 In addition, and unrelated to the cost  issue, the sentence in the introduction:

 “To date, there have been no large-scale (national or regional), non-donor funded implementations of *Wolbachia* replacement or suppression approaches.” is factually incorrect. As of today there are large government funded replacement programs running across multiple cities in both Brazil and Indonesia that are not donor funded. This should be updated or deleted.

Is the work clearly and accurately presented and does it cite the current literature?

Partly

If applicable, is the statistical analysis and its interpretation appropriate?

I cannot comment. A qualified statistician is required.

Are all the source data underlying the results available to ensure full reproducibility?

Yes

Is the study design appropriate and is the work technically sound?

Partly

Are the conclusions drawn adequately supported by the results?

Partly

Are sufficient details of methods and analysis provided to allow replication by others?

No

Reviewer Expertise:

Wolbachia biology and its use for disease control

I confirm that I have read this submission and believe that I have an appropriate level of expertise to confirm that it is of an acceptable scientific standard, however I have significant reservations, as outlined above.

# Response

**Published:** 2024-10-14

**Authors:** Katie Tiley

Thank you for reviewing this manuscript. Exactly as suggested, the manuscript has now been edited throughout to remove statements indicating that a combined suppress and then release programme presents cost savings. Additionally, the outdated statement about the lack of donor-funded implementations has now been removed. We hope you now find this manuscript acceptable for publication.

# Reviewer report: Reviewer response for version 1

**DOI:** 10.21956/gatesopenres.15611.r34325

**Published:** 2023-08-03

**Authors:** Perran Ross

**Affiliations:** 1 The University of Melbourne, Melbourne, Victoria, Australia

General comments

 Mosquitoes infected with Wolbachia bacteria are being used to control the spread of arboviruses including dengue. These programmes involve the release of lab-reared mosquitoes at a large scale into the natural population. After reaching a high frequency, the Wolbachia infection can be self-sustaining in the mosquito population due to its maternal transmission and ability to induce cytoplasmic incompatibility, and this can provide ongoing protection against dengue. The World Health Organization (WHO) has recently drafted a Target Product Profile (TPP) which includes a set of desired characteristics for Wolbachia strains and release programmes that would be required to meet WHO targets of reducing the global burden of dengue by 25%

 This manuscript addresses aspects of the TPP using models comparing variables that affect the success of Wolbachia population replacement and mapping of locations where Wolbachia releases are likely to provide the most benefit. While there is value in the latter, I have concerns with their Wolbachia replacement models and hybrid “suppress then replace” models. The Wolbachia replacement model does not provide information that isn’t already clear from previous modelling studies and analysis of the field trials, while the hybrid suppression then replace component makes inappropriate comparisons between different suppression interventions and does not consider the potentially substantial costs of these interventions.

 In the first part of the paper, the authors model Wolbachia infection frequencies and mosquito population sizes when there are different numbers of releases. Unsurprisingly, more releases and higher release ratios equals faster population replacement and a temporarily higher number of adult mosquitoes. The importance of these variables has been taken into account when planning releases and they have also been addressed in other models (e.g. Hancock et al. 2011a and 2011b). Another outcome of the model is that complete Wolbachia coverage can be achieved in under a year, but this has already been demonstrated directly through several field trials, so it is unclear what value the model here adds.

 The authors emphasize the importance of balancing the speed of Wolbachia replacement with the potentially negative outcomes of temporarily increasing the mosquito population. However, I am unsure if this is supported by the outcomes of their models, which show a maximum temporary increase in the population size of under 20%. Is this likely to be noticeable by the community? And even with this increase, won’t there be significantly more nuisance biting at other times of the year (assuming that Wolbachia releases take place when mosquito populations are low)? If this increase is likely to be a concern, then surely the use of a hybrid suppress then replace approach, which the authors discuss later in the paper, would also be a concern due to the much higher release ratios required for releases of incompatible or sterile males. While males don’t bite, they can also be regarded as a nuisance and many people will not be able to distinguish between the sexes.

 The final set of results addresses the utility of a hybrid “suppress then replace” approach, where the mosquito population is suppressed through different tools (insecticides, sterile male releases etc.) prior to population replacement releases. This will make population replacement easier as there are fewer mosquitoes to replace. Previous studies have used this approach, for example, the very first releases of wMel involved prior suppression of the population through the removal of larval habitats (Hoffmann et al. 2011). But a key question is cost effectiveness. The authors perform a cost analysis and conclude that a hybrid approach could be cost saving, but they don’t include the cost of the suppression itself for any of the approaches being compared, making their conclusions baseless. Some of the approaches they compare can be quite expensive in their own right. The incompatible insect technique for instance requires sex sorting of mosquitoes which is very labour intensive if done mechanically or very expensive if using automated systems, and mosquitoes need to be reared at large scales to achieve suppression.

 The authors then use models to test the effect of different suppression interventions on mosquito population sizes, and later, the effect on the release ratio of Wolbachia-infected mosquitoes required for successful Wolbachia establishment. These approaches have different effects (e.g. adulticides kill off adults quickly but the population bounces back quickly, while incompatible/sterile insect approaches have slower but longer term population suppression), which is a reasonable point to make. But the authors then compare these approaches directly using literature estimates of efficacy. Parameters were taken from a single study for each approach and these studies are in no way comparable to each other. They were in different environments with different populations of mosquitoes and were done at different geographic scales, time periods and intensities. There are also issues with using a single study to represent the expected efficacy for a typical suppression programme. I just don't see how it's reasonable to compare these approaches directly and conclude that  one is more effective when the approaches have been applied at different intensities and durations.

 In summary, while the paper addresses an important issue, in my view the entomological models are problematic and/or uninformative, and the paper is not suitable for indexing without substantial fundamental changes.

**Specific comments**

 Abstract - results – These quantitative thresholds are adjusted to data from the wMel releases in Wolbachia and they should not be extrapolated to Wolbachia releases in other locations, given that Wolbachia releases can have vastly different outcomes depending on the environment

 Abstract – results - Suppression interventions will reduce the number of mosquitoes required for replacement, but this statement ignores the very high numbers of mosquitoes that are needed for the suppression itself (if using incompatible males, which the authors conclude is the most effective approach).

 Intro paragraph 2 – Not all mosquitoes infected with Wolbachia show reduced virus dissemination- be more specific about the species and Wolbachia strain.

 Intro paragraph 3  – The paragraph is a bit of an oversimplification- Wolbachia doesn’t always reach fixation even if very high frequencies are reached, for instance, due to maternal transmission failure. There is also now evidence from field trials showing that Wolbachia frequencies can fluctuate seasonally or even decline to zero even after reaching near-fixation depending on the environment.

 Intro paragraph 4 – “widespread, long-term effectiveness” is true in some locations but there are also cases where Wolbachia releases have failed (see above), meaning that there are likely to be environmental constraints on where Wolbachia infections can successfully establish

 Methods – entomological model paragraph 3 – this seems to only cover a scenario where Wolbachia-infected mosquitoes are released as eggs. Please provide some context in the introduction as egg releases are not mentioned before here. The models are built on the assumption of egg releases, and the fact that Wolbachia-infected larvae are initially separate from wild larvae is an important component, but there is no justification for this or acknowledgement of other types of release. The authors discuss population suppression through male releases, but this will require adult releases.

 Methods- suppression – “The efficacy of each method was based on evidence sourced from the literature” - this section is quite subjective and there is no information about how the authors searched for studies or selected them aside from having a preference for large randomised controlled studies.

 Methods – suppression – I would prefer if the authors didn’t use “larvicides” here- guppies are not larvicides – they are predators. “Larvicides” typically refers to chemical insecticides or bacterial pathogens like Bt.

 Figure 1 – The model shows that the mosquito population size permanently decreases after Wolbachia establishment, but I’m not aware of any evidence for this from field release data. For instance, data from the releases in Yogyakarta shows that Wolbachia releases had minimal impact on the population size: https://journals.plos.org/plosntds/article?id=10.1371/journal.pntd.0010284

 Supp figure 1 – I’m not sure why the authors only considered costs up to 20% - data from release programs have estimated costs of around 30% for wMel (Hoffmann et al. 2011) and this is likely to vary substantially depending on the environment.

 Table S1 – Why is SIT included here if the authors found no studies to base its efficacy on? It is true that few SIT release programmes in Ae. aegypti have been published, but there is at least one (e.g. de Castro Poncio et al. 2021)

Is the work clearly and accurately presented and does it cite the current literature?

Partly

If applicable, is the statistical analysis and its interpretation appropriate?

No

Are all the source data underlying the results available to ensure full reproducibility?

Yes

Is the study design appropriate and is the work technically sound?

No

Are the conclusions drawn adequately supported by the results?

No

Are sufficient details of methods and analysis provided to allow replication by others?

Yes

Reviewer Expertise:

I am actively involved in research on Wolbachia population replacement programmes in Aedes mosquitoes, including lab experiments and analysis of field data. My comments focus on Wolbachia and mosquito biology and the outcomes of release programmes. I am not a statistician and am not qualified to comment on the equations behind the models, only their biological relevance.

I confirm that I have read this submission and believe that I have an appropriate level of expertise to state that I do not consider it to be of an acceptable scientific standard, for reasons outlined above.

## References

[1] : Strategies for introducing Wolbachia to reduce transmission of mosquito-borne diseases. *PLoS Negl Trop Dis* .2011;5(4) : 10.1371/journal.pntd.0001024 e1024 10.1371/journal.pntd.0001024 21541357 PMC3082501

[2] : Population dynamic models of the spread of Wolbachia. *Am Nat* .2011;177(3) : 10.1086/658121 323-33 10.1086/658121 21460541

[3] : Successful establishment of Wolbachia in Aedes populations to suppress dengue transmission. *Nature* .2011;476(7361) : 10.1038/nature10356 454-7 10.1038/nature10356 21866160

[4] : Novel Sterile Insect Technology Program Results in Suppression of a Field Mosquito Population and Subsequently to Reduced Incidence of Dengue. *J Infect Dis* .2021;224(6) : 10.1093/infdis/jiab049 1005-1014 10.1093/infdis/jiab049 33507265

# Response

**Published:** 2024-04-18

**Authors:** Katie Tiley

**2.1. General comments:**

***2.1.1. **Mosquitoes infected with Wolbachia bacteria are being used to control the spread of arboviruses including dengue. These programmes involve the release of lab-reared mosquitoes at a large scale into the natural population. After reaching a high frequency, the Wolbachia infection can be self-sustaining in the mosquito population due to its maternal transmission and ability to induce cytoplasmic incompatibility, and this can provide ongoing protection against dengue. The World Health Organization (WHO) has recently drafted a Target Product Profile (TPP) which includes a set of desired characteristics for Wolbachia strains and release programmes that would be required to meet WHO targets of reducing the global burden of dengue by 25%.*

**2.1.1.** We thank the reviewer for the time taken to read and thoroughly respond to this work which informed the World Health Organisation (WHO) Target Product Profile (TPP) and Preferred Product Characteristics (PPC) documents for *Wolbachia* replacement technology contributing to the goal of reaching the WHO targets of reducing global burden of dengue by 25%.

***2.1.2. **This manuscript addresses aspects of the TPP using models comparing variables that affect the success of Wolbachia population replacement and mapping of locations where Wolbachia releases are likely to provide the most benefit. While there is value in the latter, I have concerns with their Wolbachia replacement models and hybrid “suppress then replace” models. The Wolbachia replacement model does not provide information that isn’t already clear from previous modelling studies and analysis of the field trials, while the hybrid suppression then replace component makes inappropriate comparisons between different suppression interventions and does not consider the potentially substantial costs of these interventions.*

**2.1.2. **We agree with the reviewer that previous modelling studies and field trials have reported similar findings to our *Wolbachia* replacement model. We consider our model outcomes being consistent with these previous published findings to be a strength, not a weakness, as this provides supporting evidence for the appropriateness of our methodology, and this supported the decision of the Technical Advisory Group (TAG) to include these specific criteria in the TPP. Specifically, the value of our *Wolbachia* replacement model and hybrid suppress-then-replace model is in quantifying the differences in mosquito population dynamics compared across different variables, such as different levels of release ratio or release number. Quantifying these measures is important because it allows us to link different scenarios to cost estimates and enables more informed decisions when addressing trade-offs between different scenarios, both essential for producing reliable values for the TPP and PPC documents.

 Furthermore, we would like to make clear that the hybrid suppress-then-replace scenarios are presented to enable public health departments and organisations to better integrate their current vector control efforts with a novel *Wolbachia* replacement programme. These scenarios demonstrate the potential benefits of leveraging vector control interventions that are already in use (or could be with minor adaptations) during the transition to *Wolbachia*replacement and we acknowledge that the additional cost of building the capabilities to implement new methods of suppression solely for the purpose of reducing the cost of *Wolbachia* replacement is unlikely to reduce overall cost. Since current vector control practices are already budgeted for in routine vector control programmes, we assume no additional cost for using conventional vector control methods prior to implementing *Wolbachia* replacement. That the suppression scenarios outlined in our study are most applicable to circumstances where suppression is already in place has been further clarified in the fifth paragraph of the results section under the subheading “Exploring the dynamics of a hybrid ‘suppress then replace’ approach”: 

*“…than a hybrid programme will cost less than replacement alone. This might refer to specific circumstances when suppression might be achieved at negligible cost, for example, if it’s already part of a vector control programme. These are ideal situations where the resources for suppression are already in place, however, in most cases the potential savings achieved by a hybrid suppress then replace programme won’t justify the additional cost of implementing a novel suppression programme.”*

***2.1.3.** In the first part of the paper, the authors model Wolbachia infection frequencies and mosquito population sizes when there are different numbers of releases. Unsurprisingly, more releases and higher release ratios equals faster population replacement and a temporarily higher number of adult mosquitoes. The importance of these variables has been taken into account when planning releases and they have also been addressed in other models (e.g. Hancock et al. 2011a and 2011b). Another outcome of the model is that complete Wolbachia coverage can be achieved in under a year, but this has already been demonstrated directly through several field trials, so it is unclear what value the model here adds.*

**2.1.3.** Again, we consider the consistency of our findings with the existent literature to be an advantage, especially when considering fundamental dynamics mentioned by the reviewer; specifically, that more releases and higher release ratios leading to faster population replacement and that population replacement is achievable in under a year (e.g. Hancock et al, 2011a, 2011b). This is also the case with our finding that *Wolbachia* replacement can cause mosquito population exacerbation, previously modelled by Yakob et al (2017). The strength of this evidence from a variety of methods has been further clarified in the manuscript discussion: 

*“The population dynamics shown in our model are consistent with previous modelling and field studies showing temporary population exacerbation and successful fixation within one year (44-46); this evidence supports the credibility of our findings quantifying the difference between scenarios with varying release ratio and release number.”*

 This manuscript aims to highlight the value modelling specifically adapted to the questions raised by the TAG in the TPP process can bring. These questions inevitably span a range of field, laboratory and data analysis studies which are also cited as evidence for the criteria that are set. The additional value of the bespoke modelling we did for this showed the trade-offs between criteria, e.g. yes replacement is achievable within 6 months, but what release ratios are necessary to achieve this and what consequences do they have for cases and community acceptability?

***2.1.4. **The authors emphasise the importance of balancing the speed of Wolbachia replacement with the potentially negative outcomes of temporarily increasing the mosquito population. However, I am unsure if this is supported by the outcomes of their models, which show a maximum temporary increase in the population size of under 20%. Is this likely to be noticeable by the community? And even with this increase, won’t there be significantly more nuisance biting at other times of the year (assuming that Wolbachia releases take place when mosquito populations are low)? If this increase is likely to be a concern, then surely the use of a hybrid suppress then replace approach, which the authors discuss later in the paper, would also be a concern due to the much higher release ratios required for releases of incompatible or sterile males. While males don’t bite, they can also be regarded as a nuisance and many people will not be able to distinguish between the sexes.*

**2.1.4.** Any temporary population increase has the potential to decrease rates of community acceptance and undermine community engagement. This was an issue specifically raised as a concern by the TAG with our models quantifying the trade-offs between faster time to fixation but with higher temporary exacerbation when release ratios are increased. The temporary population increase shown in our model could be considered small (maximum exacerbation 20%) but it is important to quantify these levels for obtaining informed consent from communities. Furthermore, despite not being modelled here, even temporary minor increases could be epidemiologically important if *Wolbachia* is implemented at times of high transmission intensity, e.g. as an outbreak response. We agree with the reviewer that some suppression techniques would require additional mosquito releases, such as the male *Wolbachia*release or sterile insect technique, however, these would be accompanied by substantial separate community engagement activities and, since we recommend the hybrid suppress-then-release scenarios to complement existing vector control activities, likely already have taken place ahead of implementing *Wolbachia* replacement. Specifically, building community support through education campaigns would involve emphasising that males do not bite, as the reviewer comments, and that the exacerbation will not increase risk of infection. This has been further clarified in the manuscript: 

*“The mosquito-release suppression techniques (i.e., 1gSLT, SIT, Male Wolbachia release) may also lead to a temporary increase in mosquito population and would require separate community engagement activities to emphasise that the released males do not bite or increase the risk of infection.”*

***2.1.5.** The final set of results addresses the utility of a hybrid “suppress then replace” approach, where the mosquito population is suppressed through different tools (insecticides, sterile male releases etc.) prior to population replacement releases. This will make population replacement easier as there are fewer mosquitoes to replace. Previous studies have used this approach, for example, the very first releases of wMel involved prior suppression of the population through the removal of larval habitats (Hoffmann et al. 2011). But a key question is cost effectiveness. The authors perform a cost analysis and conclude that a hybrid approach could be cost saving, but they don’t include the cost of the suppression itself for any of the approaches being compared, making their conclusions baseless. Some of the approaches they compare can be quite expensive in their own right. The incompatible insect technique for instance requires sex sorting of mosquitoes which is very labour intensive if done mechanically or very expensive if using automated systems, and mosquitoes need to be reared at large scales to achieve suppression.*

**2.1.5.** We acknowledge the reviewers concern regarding the cost of suppression, and our conclusions around cost savings previously recognise this depends on the cost of suppression, for example in the results: 

*”All hybrid programmes had the potential to offer cost savings over replacement alone, depending on the cost of suppression… This, however, does not take into account the costs of suppression. A different interpretation of these results would be: if suppression can be achieved for less than these costs, then a hybrid programme will cost less than replacement alone.”*

 And in the discussion: 

*“We predict that insect-based suppression methods (1gSLT, SIT, Male Wolbachia release) will be more effective than conventional suppression tools, but also outline a limited cost window which may be challenging for insect-based suppression methods to achieve. Investment in new infrastructure to conduct insect-based suppression may not be justified for a one-off suppression, but between overlapping resource requirements for suppression and replacement, ongoing use post-replacement (e.g., outbreak control or to achieve dengue elimination) and a continued drive to lower costs of mosquito suppression (52), this investment cost may be justified.”*

 However, we understand that expressing the hybrid approach as cost saving has the potential to be misinterpreted and therefore, as mentioned in point 2.1.2, have further edited the results to better clarify our conclusions.

***2.1.6.** The authors then use models to test the effect of different suppression interventions on mosquito population sizes, and later, the effect on the release ratio of Wolbachia-infected mosquitoes required for successful Wolbachia establishment. These approaches have different effects (e.g. adulticides kill off adults quickly but the population bounces back quickly, while incompatible/sterile insect approaches have slower but longer term population suppression), which is a reasonable point to make. But the authors then compare these approaches directly using literature estimates of efficacy. Parameters were taken from a single study for each approach and these studies are in no way comparable to each other. They were in different environments with different populations of mosquitoes and were done at different geographic scales, time periods and intensities. There are also issues with using a single study to represent the expected efficacy for a typical suppression programme. I just don't see how it's reasonable to compare these approaches directly and conclude that  one is more effective when the approaches have been applied at different intensities and durations.*

**2.1.6.** We appreciate the reviewer’s comments regarding the difficulties encountered when comparing different suppression interventions. A WHO Evidence Review Group is currently conducting a rigorous systematic review of *Aedes* mosquito control effectiveness that was unfortunately not available at the time of analysis and remains in development. In the absence of this we aimed to derive evidence-based scenarios for suppression effectiveness which allowed approximation of the differences between methods of suppression at 5- and 10-week periods. We acknowledge that building the evidence base for these scenarios is not always straightforward to extract from the literature due to different study designs and contexts. To mitigate this complexity in our models we also include a range of hypothetical fixed effectiveness values for direct comparison between different suppression methods, this has been clarified in the discussion of limitations: 

*“Additionally, suppression efficacy is highly context-dependent; to mitigate this we modelled an hypothetical 20%, 50%, and 80% suppression efficacy to allow clearer comparison between suppression techniques.”*

 Our main findings (ie that insect release strategies offer advantages over conventional suppression strategies) are the same whether using the literature-derived efficacy estimates or the fixed efficacies. Another advantage of using a range of fixed efficacies is that it allows readers or implementers to interpret these results in different contexts where higher or lower suppression effectiveness may be feasible.

***2.1.7.** In summary, while the paper addresses an important issue, in my view the entomological models are problematic and/or uninformative, and the paper is not suitable for indexing without substantial fundamental changes.*

**2.1.7.** We are glad the reviewer agrees that this modelling work informs decisions in an important area of research. We hope that the above general responses and below specific responses clarify the value of the entomological models and their importance for this specific application of informing the *Wolbachia* replacement TPP and hybrid suppress then replace PPC.

**2.2. Specific comments:**

***2.2.1.** Abstract - results – These quantitative thresholds are adjusted to data from the wMel releases in Wolbachia and they should not be extrapolated to Wolbachia releases in other locations, given that Wolbachia releases can have vastly different outcomes depending on the environment*

**2.2.1.** The statement: 

*“We estimate that for Wolbachia replacement to be deployable in enough areas to make major contributions to reducing global dengue burden by 25% (in line with 2030 WHO targets), it must have the potential for cost be reduced to between $7.63 and $0.24 (USD) per person protected or less”*

 Does not depend on any data from wMel releases. These figures come directly from table 2 with a legend:

*“The predicted target cost per person for Wolbachia replacement based on different assumptions about desired global impact (rows) and averted medical and outbreak control costs (assumed proxy of willingness to pay, columns).”*

 These figures are based on the cost of illness and conventional control alone, while effectiveness was set by the TAG for the TPP at an assumed 70%, neither of which depend on data from the *wMel* releases.

***2.2.2.** Abstract – results - Suppression interventions will reduce the number of mosquitoes required for replacement, but this statement ignores the very high numbers of mosquitoes that are needed for the suppression itself (if using incompatible males, which the authors conclude is the most effective approach).*

**2.2.2.** This statement refers to how suppression reduces the number of mosquitoes required during *Wolbachia*replacement. This was identified as a key parameter by the TAG because it requires release of biting, potentially dengue-transmitting, mosquitoes and therefore has a different set of community concerns, engagement strategies, costs and regulatory implications to the release of male mosquitoes. We also clarify in various sections of the manuscript that the suppression in hybrid suppress-then-replace strategies is recommended to complement existing vector control strategies, therefore, releasing mosquitoes for insect-release suppression technologies (i.e., Male *Wol*release, SIT, and 1gSLT) would already be in effect, meaning that there would be a net reduction in released mosquitoes (both male and female) in such a hybrid strategy.

***2.2.3.** Intro paragraph 2 – Not all mosquitoes infected with Wolbachia show reduced virus dissemination- be more specific about the species and Wolbachia strain.*

**2.2.3. **We agree it was not clear from the text that not all *Wolbachia* strains reduce virus dissemination, therefore this has been clarified in text: 

*“Ae. aegypti mosquitoes infected with* *certain strains of the bacterium Wolbachia, such as wMel, wMelPop, and wAlbB (7)* *show reduced rates of virus dissemination, making them less capable of transmitting arboviruses (8).”*

***2.2.4. **Intro paragraph 3  – The paragraph is a bit of an oversimplification- Wolbachia doesn’t always reach fixation even if very high frequencies are reached, for instance, due to maternal transmission failure. There is also now evidence from field trials showing that Wolbachia frequencies can fluctuate seasonally or even decline to zero even after reaching near-fixation depending on the environment.*

**2.2.4. **We agree that *Wolbachia* is not always successful reaching fixation and has been known to fluctuate or decline. That environmental variation affects *Wolbachia* replacement dynamics has been clarified in the text: 

*“Modelling has shown that once a critical proportion of mosquitoes in the population have Wolbachia, coverage should continue to increase to fixation without further releases, but below this threshold Wolbachia coverage may decline (possibly to zero) once releases stop due to fitness costs associated with released mosquito strains ^8 ^… It should be noted that in practice, Wolbachia frequencies may fluctuate seasonally and still decline to zero after reaching fixation depending on environmental variables such as temperature, rainfall, and physical barriers (12, 13).”*

***2.2.5. **Intro paragraph 4 – “widespread, long-term effectiveness” is true in some locations but there are also cases where Wolbachia releases have failed (see above), meaning that there are likely to be environmental constraints on where Wolbachia infections can successfully establish*

**2.2.5. **We further acknowledge the need to emphasise there is heterogeneity in the success of *Wolbachia*replacement, and have edited the text to underscore these considerations: 

*“A growing range of entomological, epidemiological and modelling evidence supports the widespread, long-term effectiveness of Wolbachia replacement (14-16), and research continues to identify environmental conditions associated with spatially and temporally heterogeneous Wolbachia establishment (13).”*

***2.2.6. **Methods – entomological model paragraph 3 – this seems to only cover a scenario where Wolbachia-infected mosquitoes are released as eggs. Please provide some context in the introduction as egg releases are not mentioned before here. The models are built on the assumption of egg releases, and the fact that Wolbachia-infected larvae are initially separate from wild larvae is an important component, but there is no justification for this or acknowledgement of other types of release. The authors discuss population suppression through male releases, but this will require adult releases.*

**2.2.6.** We appreciate the reviewers feedback that the text was unclear about the different methods of mosquito release during *Wolbachia* replacement. Our work modelled egg releases in release cups only; text has been added to the entomological model section of the methods for additional context: 

*“Since this model was calibrated using data of Wolbachia fixation dynamics from the Yogyakarta RCT (16), any features of this RCT influenced the methodology, such as delivery mode (egg releases in release cups) and the number of releases (ranging from nine to 14), therefore, other delivery modes such as releasing Wolbachia-infected adult Ae. aegypti were not simulated.”*

 Each of the insect-release suppression technologies (Male *Wol* release, SIT, and 1gSLT) were simulated with adult releases.

***2.2.7. **Methods- suppression – “The efficacy of each method was based on evidence sourced from the literature” - this section is quite subjective and there is no information about how the authors searched for studies or selected them aside from having a preference for large randomised controlled studies.*

**2.2.7. **We acknowledge the literature-derived efficacies have limitations, such as estimates of effectiveness being context specific, variations in implementation and ambiguous reporting of effectiveness values. It is precisely because of this that we did not pursue a systematic search for studies with specific inclusion or exclusion criteria. Instead, we chose studies that we judged best evaluated the effectiveness of short-term pulses of the intervention. We clearly cite the sources of these assumed literature-derived effectiveness values and acknowledge that readers may expect alternative values in different settings. To address this, we chose to also include a range of hypothetical values (20%, 50%, and 80%) for suppression effectiveness which allow a more standardised comparison of the dynamics of different methods of suppression and how such dynamics affect requirements for successful replacement programmes. As mentioned earlier, this has been further emphasised in the discussion of limitations.

*“Additionally, suppression efficacy is highly context-dependent; to mitigate this we modelled an hypothetical 20%, 50%, and 80% suppression efficacy to allow clearer comparison between suppression techniques.”*

***2.2.8.** Methods – suppression – I would prefer if the authors didn’t use “larvicides” here- guppies are not larvicides – they are predators. “Larvicides” typically refers to chemical insecticides or bacterial pathogens like Bt.*

**2.2.8. **Since both chemical larvicides and guppies primary mode of action is to kill eggs, larvae and pupae, their effects on the model are identical, therefore we believe the hypothetical range of values (20%, 50%, and 80%) can refer to either larvicides, which are more widely used, or guppies. However, in response to this comment the text has been edited to clarify this labelling specifically: 

*“Larvicides were simulated by equally reducing the number of eggs, larvae, and pupae. Because the effect is similar and because the best measurement of effectiveness of an intervention that targets the aquatic stages of the mosquito came from a trial of predatory guppies, this effectiveness as measured by Hustedt et al (37) was chosen to represent the effectiveness of larvicides.”*

***2.2.9.** Figure 1 – The model shows that the mosquito population size permanently decreases after Wolbachia establishment, but I’m not aware of any evidence for this from field release data. For instance, data from the releases in Yogyakarta shows that Wolbachia releases had minimal impact on the population size: https://journals.plos.org/plosntds/article?id=10.1371/journal.pntd.0010284*

**2.2.9. **We assumed that *Wolbachia* exerts a fitness cost, following the reported evidence (e.g., Joubert et al, 2016; Ross & Hoffmann, 2022), and as a consequence of this, the total population size was reduced following replacement with *Wolbachia*-infected mosquitoes. The fitness cost we selected was intended as a conservative estimate to reduce risk of under-estimation in field deployments, so the projected reduction in total mosquito numbers is thus likely a concomitant over-estimation.

***2.2.10.** Supp figure 1 – I’m not sure why the authors only considered costs up to 20% - data from release programs have estimated costs of around 30% for wMel (Hoffmann et al. 2011) and this is likely to vary substantially depending on the environment.*

**2.2.10.** We agree that the fitness cost of *Wolbachia* infection is very dependent on the environment. We edited the manuscript to emphasise this:

*“Due to the fitness cost of Wolbachia (conservatively modelled to be 20% (39), but highly variable depending on environment (41))...”*

 Furthermore, we have extended the range of values included in the sensitivity analysis in Supplementary Figure 1 to encompass the 30% value highlighted by the reviewer.

***2.2.11.** Table S1 – Why is SIT included here if the authors found no studies to base its efficacy on? It is true that few SIT release programmes in Ae. aegypti have been published, but there is at least one (e.g. de Castro Poncio et al. 2021)*

**2.2.11.** The lack of a literature-derived efficacy value for SIT suppression technique was a genuine oversight and we’re grateful for the reviewer’s feedback; the manuscript has been amended to include a literature-derived efficacy for SIT. A literature-derived efficacy of approximately 49% at 5-weeks and 77% at 10-weeks has been extracted from the field trial by De Castro Poncio et al (2021) Figure 2b. The analyses which previously used an hypothetical 50% efficacy for this suppression technique have been updated to use the literature-derived 49% efficacy, correspondingly there has been only marginal changes in resulting population dynamics and no change in the overall trends or findings discussed. The methods section for SIT suppression have been updated to include this development:

*“Parameters for the hypothetical fixed rate efficacy of 20%, 50%, and 80% were calculated by comparing the total adult population at model equilibrium with the minimum adult population reached after five weeks of application. The literature-derived efficacy values were 49% five weeks after the last suppression period and 77% ten weeks after the last suppression period (33), calculated by comparing the total adult population at model equilibrium to the total adult population after five- or ten-weeks of suppression which achieved the desired efficacy (summarised in Supplementary Table 1). A caveat of this approach is that the resulting minimum adult population is reached later than five- or ten-weeks, therefore, the maximum efficacy calculated in these scenarios is marginally greater than the literature value stated (shown in Supplementary Figure 2).”*

 In addition, SIT values have been updated in Figures 4, 5, and 6, added to Supplementary Figure 2, and the SIT literature source has been added to Supplementary Table 1.

**References**

 De Castro Poncio, L., Dos Anjos, F. A., De Oliveira, D. A., Rebechi, D., De Oliveira, R. N., Chitolina, R. F., Fermino, M. L., Bernardes, L. G., Guimarães, D., Lemos, P. A., Silva, M. N. E., Silvestre, R. G. M., Bernardes, E. S., & Paldi, N. (2021). Novel Sterile Insect Technology Program Results in Suppression of a Field Mosquito Population and Subsequently to Reduced Incidence of Dengue. *Journal of Infectious Diseases*, *224*(6), 1005–1014. https://doi.org/10.1093/infdis/jiab049

 Hancock, P. A., Sinkins, S. P., & Godfray, H. C. J. (2011a). Population dynamic models of the spread of Wolbachia. *American Naturalist*, *177*(3), 323–333. https://doi.org/10.1086/658121

 Hancock, P. A., Sinkins, S. P., & Godfray, H. C. J. (2011b). Strategies for introducing Wolbachia to reduce transmission of mosquito-borne diseases. *PLoS Neglected Tropical Diseases*, *5*(4). https://doi.org/10.1371/journal.pntd.0001024

 Joubert, D. A., Walker, T., Carrington, L. B., De Bruyne, J. T., Kien, D. H. T., Hoang, N. L. T., Chau, N. V. V., Iturbe-Ormaetxe, I., Simmons, C. P., & O’Neill, S. L. (2016). Establishment of a Wolbachia Superinfection in Aedes aegypti Mosquitoes as a Potential Approach for Future Resistance Management. *PLoS Pathogens*, *12*(2). https://doi.org/10.1371/journal.ppat.1005434

 Ross, P. A., & Hoffmann, A. A. (2022). Fitness costs of Wolbachia shift in locally-adapted Aedes aegypti mosquitoes. *Environmental Microbiology*, *24*(12), 5749–5759. https://doi.org/10.1111/1462-2920.16235

 Yakob, L., Funk, S., Camacho, A., Brady, O., & Edmunds, W. J. (2017). Aedes aegypti Control Through Modernized, Integrated Vector Management. *PLoS Currents*, 1–34. https://doi.org/10.1371/currents.outbreaks.45deb8e03a438c4d088afb4fafae8747

# Reviewer report: Reviewer response for version 1

**DOI:** 10.21956/gatesopenres.15611.r34088

**Published:** 2023-08-03

**Authors:** Scott O'neill

**Affiliations:** 1 World Mosquito Program, Monash University, Clayton, Victoria, Australia

This paper uses entomological and economic modeling approaches to derive programmatic cost targets to support a WHO defined TPP for *Wolbachia *replacement. The starting premise for the modeling approach is that *Wolbachia *replacement needs to be deployable on a global scale at a sufficiently low cost to achieve the WHO’s goal of a 25% reduction in global dengue incidence by 25% (relative to a 2010-2020 baseline). This premise, and the consequent epidemiologic and economic assumptions for how *Wolbachia *could be most cost-effectively deployed at a global scale, lead to overly simplistic conclusions around a target global cost of deployment of *Wolbachia *replacement. Although the authors have qualified this by stating that “TPP targets represent the lowest averted medical and outbreak control costs per person among all areas where releases are required, there are many areas that could support higher programme costs”, the unrealistic target of reducing 25% of national dengue burden and averting these costs against the lowest medical and outbreak control costs over three years provides an overly optimistic global PCC of $0.24 per person. The linkage in this instance has a substantial impact on the total cost of coverage. The current modeling approach does not adequately address this issue. The entomological modelling of the hybrid “suppress then replace” is based largely on theoretical estimates of suppression efficacy (Figure 4) or extrapolation of data from small scale pilot studies. The assumptions behind some of these approaches are flawed, and present an unrealistic scenario around the feasibility of undertaking hybrid suppress and then replace methods. This section is highly speculative, adds little to the paper and should be removed.

 Specific comments:

 1. In order to frame the impact targets in the TPP in terms of the WHO NTDs roadmap target of a 25% reduction in global dengue burden by 2030, the authors identify the priority settings for achievement of that target by ranking 5x5km pixels globally (Page 4). There are several issues with this approach to ranking target geographies:

 1a. The authors state on page 4 that they “identified which areas (5km x 5km pixels) would need to be targeted to reach the WHO goal of reducing global dengue burden by 25% in the most net cost-efficient manner. To do this, each 5km x 5km pixel was ranked from highest to lowest based on a benefit (averted medical and outbreak costs) to cost (approximate *Wolbachia *program cost estimate based on population density from Brady et al 2020) ratio.” A critical input into this calculation of the pixel-level benefit-to-cost ratio, which forms the basis for identifying the target geographies considered in the rest of the modeling, is the assumed pixel-level cost of *Wolbachia *deployment, yet insufficient detail is provided about that assumed intervention cost. The paper cited reports an economic analysis of *Wolbachia *deployment in Indonesia, in which the predicted *Wolbachia *program cost was modeled as a cost per km2, as a function of human population density. Presumably the current paper also used cost per km2 as the input for *Wolbachia *program cost in calculating pixel-level benefit-cost ratios, and this should be described in more detail. More fundamentally, it seems circular to identify target geographies based on a benefit-to-cost ratio that requires an assumption of programmatic cost per km2 / per person as an input, and then use the predicted avertable cost of dengue in those target geographies to define a target programmatic cost of *Wolbachia *per person. Please clarify the logic of this approach, and why the inclusion of a *Wolbachia *cost assumption in the initial step of ranking the target geographies doesn’t compromise the approach.

 1b. The ranking of 5x5 km pixels to identify target geographies for *Wolbachia *deployment  produces unrealistic scenarios of fragmented small release areas (totalling 924,557 km2 in size) that are distributed across 73 different countries. This undermines the cost-per-person assumptions, as it would clearly be more costly to deploy across these fragmented areas than to cover large contiguous urban populations.To achieve the most net cost-efficient implementation of *Wolbachia *at a global scale, ranking would be better done at e.g. administrative 2 level to identify the contiguous highest-burden areas in which the global reduction targets could be achieved or, if the pixel-based approach is to be used, at least apply an additional criterion that filters out identified target release areas below a minimum km2 (e.g. 10 km2) or population size (e.g. 50,000 population). It is evident from the datasets of target release area km2 and population at 2nd level administrative units, provided in the supplementary materials, that many of the target geographies are far too small to support *Wolbachia *replacement deployments in reality.

 Despite this being one of the major limitations of the modeling approach, the authors give no consideration at all in the discussion to the fact that in real-world programmatic implementation of *Wolbachia *replacement, it will always be most efficient from operational, entomological, and cost-efficiency perspectives to deploy across a large contiguous urban area rather than small fragmented release areas. This needs to be addressed in the discussion.

 2. The 'Required cost per person covered' thresholds in Table 2 are presented at a global level, based on the projected costs averted in the least cost effective setting in any given scenario. Although regional and national cost targets are included in the supplementary materials, and the authors acknowledge in the discussion that many countries could support higher costs and ‘that a *Wolbachia *replacement product would still meet the TPP targets if initial program costs were higher and if the product has the potential to reduce costs down to the $2.33 target, this should be presented more explicitly within the primary results. It is not meaningful to report the primary cost targets at a global level without including also the range of national- and regional-level target costs, as that's not the spatial scale at which economies of scale in production and implementation costs are achieved, or at which customers' willingness-to-pay or funding decisions operate.

 3. The cost targets are very conservative in requiring cost-neutrality from a health system perspective (i.e. implementation cost ≤ direct medical costs + outbreak vector control costs averted) and assuming only 3-5 years of benefits for the preferred TPP. Although a scenario of 10 years of benefits is also included in Table 2, the authors present the cost target of $2.33 as the minimum TPP, based on a scenario of achieving a 25% global burden reduction and only 5 years of benefit, with a PPC cost target of $0.24 (25% national burden reduction in all countries and only 3 years of benefit). These durations of benefit are too pessimistic given the evidence from field studies of *Wolbachia *durability in *Ae. aegypti* populations for at least 10 years.     

 4. The entomological modelling of the hybrid “suppress then replace” is based largely on theoretical estimates of suppression efficacy (Figure 4), or extrapolation of population suppression results from small scale pilot interventions. This reviewer questions whether the efficacy of these suppression methods can ever be achieved at the scale required to avert 25% of the global dengue burden. The assumptions around the suppressive effects of a single round of intervention seem overly simplistic, and are generally not consistent with field implementations that take many rounds of intervention to produce consistent and reproducible suppression (see Supplementary Table 1 references below). “With reductions in adult mosquito population size in the range of 8.18 – 43.51%, from a single application all methods of suppression were predicted to remove the ~1–10% mosquito population exacerbation seen in replacement only programmes.” (Page 12) Further, “Our model predicts that a prior suppression programme of five weekly rounds could reduce the number of *Wolbachia *mosquitoes required to reach target coverage within 6 months by 16-81% depending on suppression method used.” (Pages 12-13)

 Based on empirical data from a Male *Wolbachia *release program that targeted *Ae. aegypti*, there is questionable evidence that a 5-week suppression program would substantially impact mosquito population abundance. In the Supplementary Table 1, it indicates a 5-week suppression efficacy of 60%, and 10-week suppression efficacy of 90% (sourced from Ching et al. 2021). This is incorrect. Figure 2, Panel G indicates <10% suppression out to week 7 in the Yishun Core Area where releases were concentrated. In the Tampines Core area, suppression efficacy varied significantly over the first 12-13 weeks, ranging from 0-40%. Ching et al. (2021) presented a statistical analysis of the suppression effect (Figure 3, Panels A and C) and found no significant suppression in Yishun until the fourth month of releases, and variable results in Tampines (statistically significant suppression in first month, but not significant in the second month of releases).

 Similarly for the “1gSLT” suppression rates of 45% and 75% at weeks 5 and 10, respectively (Supplementary Table 1. Suppression Efficacy Carvalho et al. 2015). These data were extracted from Figure 2, panel D, from the 5- and 10-week timepoints in release area A, the authors have failed to state that there were 7-months of releases undertaken across these combined Areas A and B prior to the 5- and 10-week suppression period over which the above rates were calculated (for Area A). Also of note was the very small size of the suppression area in this trial (Area A = 0.055km2). Again, the assumptions drawn from these studies are inaccurate and should be withdrawn.

 How have the SIT suppression efficacy estimates been validated? In Supplementary Table 1, it states there is no currently published trials for suppression using SIT, and also in the Methods it states “There are currently no published randomised controlled trials for suppression of *Aedes* mosquitoes using SIT, therefore 50% efficacy is shown for SIT in analyses where only literature-derived efficacy is used. What is the “literature derived efficacy”?

 The assumptions underpinning the modelling of the suppress then replace section of the paper appear to be erroneous, or at least very optimistic of their effectiveness in terms of suppression of the mosquito populations. This section is highly speculative, adds little to the paper and should be removed.

 5. “All hybrid programmes had the potential to offer cost savings over replacement alone, depending on the cost of suppression” (Page 14). This statement is not supported by presentation of any costing data for suppression. From an economic perspective, there is no evidence that a hybrid approach of suppression followed by replacement would be more cost effective than *Wolbachia *replacement alone, and a recent study based on data from Singapore’s *Wolbachia *suppression program suggests that a hybrid suppression-followed-by-replacement program could be significantly more expensive (Soh *et al*. 2021 ^1^). The current annual per capita cost of suppression in Singapore is approximately USD $5 per person reached. This cost is roughly equivalent to the one-time, all-inclusive cost required to achieve *Wolbachia *replacement across some target settings (Brady et al BMC Med 2020; Turner et al PLoS Negl Trop Dis 2023). The ‘potential cost savings enabled by a hybrid approach’ presented in Table 3 “assumes suppression has no cost or is an in-kind contribution”, which is entirely implausible in any setting, let alone in a scenario of large-scale global implementation across the 73 countries (>900,000 km2), which would be required to achieve the TPP minimum targets. Without a more extensive analysis of the efficacy of suppression programs and their cost, the current analyses are highly speculative and add little to the paper. This reviewer feels the whole section on hybrid “suppress then replace” should be removed.

**Minor comments:**

  

1. Page 3, Introduction, second paragraph: The virus-blocking phenotype is specific to the species of *Wolbachia, *mosquito, and virus - the authors should use more specific language here.
2. Page 3, Introduction, second paragraph: There is no empirical evidence that temporary increases in the *Ae. aegypti *mosquito population have been a key barrier to community acceptability of *Wolbachia *releases; this should be expressed as theoretical consideration.
3. Page 6, Results, paragraph 1 (and throughout): the terminology ‘coverage’ is misleading as it implies the % coverage of a geographic area, whereas the authors are referring to % *Wolbachia *prevalence in the local *Ae. aegypti *population. The proportion of *Wolbachia*-infected mosquitoes in the population should be considered the “percentage prevalence,” not the target coverage.
4. Page 11, Figure 3: The axes titles and figure legend needs to make explicit what time period this data relates to, in terms of the cumulative cases and costs averted *. *Presumably 5 years of benefit, since the $2.33 cost threshold is shown, however this is inconsistent with the $0.24 preferred criteria referred to in the figure legend, which relates to 3 years of benefit. And the lower horizontal dotted line appears to be at $0.39, not $0.24.
5. Page 16, Discussion: “Cost continues to be a barrier to wider adoption of *Wolbachia *replacement when its high costs but long-term benefits are compared to lower cost but short acting suppression methods”. This statement needs to reflect the fact that *Wolbachia *replacement has an evidence base for epidemiological impact while the short acting suppression methods do not.

Is the work clearly and accurately presented and does it cite the current literature?

Partly

If applicable, is the statistical analysis and its interpretation appropriate?

I cannot comment. A qualified statistician is required.

Are all the source data underlying the results available to ensure full reproducibility?

Yes

Is the study design appropriate and is the work technically sound?

Partly

Are the conclusions drawn adequately supported by the results?

Partly

Are sufficient details of methods and analysis provided to allow replication by others?

No

Reviewer Expertise:

Wolbachia biology and its use for disease control

I confirm that I have read this submission and believe that I have an appropriate level of expertise to confirm that it is of an acceptable scientific standard, however I have significant reservations, as outlined above.

## References

[1] : Economic impact of dengue in Singapore from 2010 to 2020 and the cost-effectiveness of Wolbachia interventions. *PLOS Glob Public Health* .2021;1(10) : 10.1371/journal.pgph.0000024 e0000024 10.1371/journal.pgph.0000024 36962069 PMC10021432

# Response

**Published:** 2024-04-18

**Authors:** Katie Tiley

***1.1 Summary comments:***

***1.1.1.** This paper uses entomological and economic modeling approaches to derive programmatic cost targets to support a WHO defined TPP for Wolbachia replacement. The starting premise for the modeling approach is that Wolbachia replacement needs to be deployable on a global scale at a sufficiently low cost to achieve the WHO’s goal of a 25% reduction in global dengue incidence by 25% (relative to a 2010-2020 baseline). This premise, and the consequent epidemiologic and economic assumptions for how Wolbachia could be most cost-effectively deployed at a global scale, lead to overly simplistic conclusions around a target global cost of deployment of Wolbachia replacement. Although the authors have qualified this by stating that “TPP targets represent the lowest averted medical and outbreak control costs per person among all areas where releases are required, there are many areas that could support higher programme costs”, the unrealistic target of reducing 25% of national dengue burden and averting these costs against the lowest medical and outbreak control costs over three years provides an overly optimistic global PCC of $0.24 per person. The linkage in this instance has a substantial impact on the total cost of coverage. The current modeling approach does not adequately address this issue.*

**1.1.1.** We are grateful that the reviewer read and thoroughly responded to this work which informed the World Health Organisation (WHO) Target Product Profile (TPP) and Preferred Product Characteristics (PPC) documents for *Wolbachia* replacement technology contributing to the goal of reaching the WHO targets of reducing global burden of dengue by 25%. We have carefully considered the reviewers feedback and responded point-by-point in detail below.

***1.1.2.** The entomological modelling of the hybrid “suppress then replace” is based largely on theoretical estimates of suppression efficacy (Figure 4) or extrapolation of data from small scale pilot studies. The assumptions behind some of these approaches are flawed, and present an unrealistic scenario around the feasibility of undertaking hybrid suppress and then replace methods. This section is highly speculative, adds little to the paper and should be removed.*

**1.1.2. **The criticism of the hybrid suppress-then-replace modelling justifiably covers some specific areas where the evidence supporting the effectiveness of several suppression tools could have been improved (which has now been implemented, see points 1.2.4a – e), but also covers wider conceptual issues of the value of the hybrid approach. One key aspect here is to clarify that modelling the hybrid approach was for the PPC process and not the TPP. This PPC process is designed to guide the development of novel products or strategies and are both more forward looking and less prescriptive than the TPP. The results from the modelling for these two sections should, therefore, also be interpreted within these different use contexts. We agree that the current evidence for hybrid suppress then replace strategies is limited, the modelling is intended to guide the (limited) use cases in which future improvements to suppression interventions may enable hybrid strategies.

***1.2 Specific comments:***

***1.2.1.** In order to frame the impact targets in the TPP in terms of the WHO NTDs roadmap target of a 25% reduction in global dengue burden by 2030, the authors identify the priority settings for achievement of that target by ranking 5x5km pixels globally (Page 4). There are several issues with this approach to ranking target geographies:*

*1.2.1a. The authors state on page 4 that they “identified which areas (5km x 5km pixels) would need to be targeted to reach the WHO goal of reducing global dengue burden by 25% in the most net cost-efficient manner. To do this, each 5km x 5km pixel was ranked from highest to lowest based on a benefit (averted medical and outbreak costs) to cost (approximate Wolbachia program cost estimate based on population density from Brady et al 2020) ratio.” A critical input into this calculation of the pixel-level benefit-to-cost ratio, which forms the basis for identifying the target geographies considered in the rest of the modeling, is the assumed pixel-level cost of Wolbachia deployment, yet insufficient detail is provided about that assumed intervention cost. The paper cited reports an economic analysis of Wolbachia deployment in Indonesia, in which the predicted Wolbachia program cost was modeled as a cost per km2, as a function of human population density. Presumably the current paper also used cost per km2 as the input for Wolbachia program cost in calculating pixel-level benefit-cost ratios, and this should be described in more detail. More fundamentally, it seems circular to identify target geographies based on a benefit-to-cost ratio that requires an assumption of programmatic cost per km2 / per person as an input, and then use the predicted avertable cost of dengue in those target geographies to define a target programmatic cost of Wolbachia per person. Please clarify the logic of this approach, and why the inclusion of a Wolbachia cost assumption in the initial step of ranking the target geographies doesn’t compromise the approach.*

**1.2.1a.** We thank the reviewer for raising this lack of clarity and concur the current description may be misinterpreted to be circular. This section has been edited to address this issue:

 “Next we identified which areas (5km x 5km pixels) would need to be targeted to reach the WHO goal of reducing global dengue burden by 25% in the most net cost efficient manner. For a generic environmental intervention, where cost of the intervention only depends on area covered, this would involve targeting areas with the highest density of dengue costs. However, because the cost of Wolbachia programmes have been shown to depend on the human population density and per capita GDP in the release area (27), this can change which areas are most important to prioritise from an optimal net cost perspective. To account for these variable implementation cost factors Wolbachia replacement is most cost effective if targeted to higher density, high dengue burden areas (27). We therefore identified which areas (5km x 5km pixels) would need to be targeted to reach the WHO goal of reducing global dengue burden by 25% in the most net cost-efficient manner. To do this, each 5km x 5km pixel was ranked from highest to lowest based on a benefit (averted medical and outbreak costs) to cost (approximate Wolbachia programme cost estimate based on population density and per capita GDP from Brady et al. (27)) ratio. For clarity, the approximate Wolbachia programme cost from Brady et al. (27) only affects the ranking of pixels (i.e. targeting), not the TPP target cost estimates. Cumulative averted cases were then calculated and pixel selection ended when averted cases first exceeded 25% of the global total….”

***1.2.1b.** The ranking of 5x5 km pixels to identify target geographies for Wolbachia deployment  produces unrealistic scenarios of fragmented small release areas (totalling 924,557 km2 in size) that are distributed across 73 different countries. This undermines the cost-per-person assumptions, as it would clearly be more costly to deploy across these fragmented areas than to cover large contiguous urban populations. To achieve the most net cost-efficient implementation of Wolbachia at a global scale, ranking would be better done at e.g. administrative 2 level to identify the contiguous highest-burden areas in which the global reduction targets could be achieved or, if the pixel-based approach is to be used, at least apply an additional criterion that filters out identified target release areas below a minimum km2 (e.g. 10 km2) or population size (e.g. 50,000 population). It is evident from the datasets of target release area km2 and population at 2nd level administrative units, provided in the supplementary materials, that many of the target geographies are far too small to support Wolbachia replacement deployments in reality. Despite this being one of the major limitations of the modeling approach, the authors give no consideration at all in the discussion to the fact that in real-world programmatic implementation of Wolbachia replacement, it will always be most efficient from operational, entomological, and cost-efficiency perspectives to deploy across a large contiguous urban area rather than small fragmented release areas. This needs to be addressed in the discussion.*

**1.2.1b.** We recognise and agree that many additional considerations will go into which areas will ultimately be targeted for *Wolbachia* implementation and the resulting map is not intended to be prescriptive, but rather give an indication of the kinds of areas that would be most sensible to prioritise from a net cost efficiency perspective. Even if the decision to implement occurs at administrative unit-level, not everywhere within an administrative unit will see *Wolbachia* releases and the pixel-level maps can be useful for informing within-administrative unit targeting subject to a set of criteria that should be locally determined. It should also be emphasised that the TPP is, by definition, for a hypothetical *Wolbachia* replacement product, not the current most widely-used *wMel* programme. This means that such considerations may differ in their importance and, as such, are beyond the scope of the TPP and the accompanying modelling analysis. We have added the following sentence to third paragraph of the discussion to convey this:

*“It is important to clarify that these maps should not be used prescriptively, but rather give an indication of the kinds of areas that are likely to be most cost efficient to target, with the final decision on which areas are targeted for release subject to additional operational, entomological, financial and political considerations.”*

 The administrative-unit summary provided in the supplement shows that 85.5% of 2nd administrative units (admin2s) have predicted release areas at or above 10km ^2^. Among the remaining admin2s with smaller release areas all of them are in countries that also contain admin2s with larger (>10km ^2^) release areas and 66% of smaller release admin2s occur in Vietnam, Colombia, Thailand, Brazil and Mexico which would all require large release areas across their respective countries. These smaller areas could thus be considered an extension of programmes in nearby areas. Introducing restrictions on contiguity or release area will, therefore, only affect a very small number of areas and have minimal impact on the overall findings.

***1.2.2. **The 'Required cost per person covered' thresholds in Table 2 are presented at a global level, based on the projected costs averted in the least cost effective setting in any given scenario. Although regional and national cost targets are included in the supplementary materials, and the authors acknowledge in the discussion that many countries could support higher costs and ‘that a Wolbachia replacement product would still meet the TPP targets if initial program costs were higher and if the product has the potential to reduce costs down to the $2.33 target, this should be presented more explicitly within the primary results. It is not meaningful to report the primary cost targets at a global level without including also the range of national- and regional-level target costs, as that's not the spatial scale at which economies of scale in production and implementation costs are achieved, or at which customers' willingness-to-pay or funding decisions operate.*

**1.2.2. **The primary purpose of this work was to support the TPP. TPPs are used to decide if particular products should or should not be developed. For this TPP the Technical Advisory Group (TAG) decided that these criteria should be set to be globally relevant – i.e. the product should only be developed if it can be globally relevant – for equity reasons. The TPP uses the specific wording that a product “must have the potential to reach” these thresholds. We have now updated the abstract and the results sections to use this specific phrase for consistency. The pathway to achieving this with initially higher costs is mentioned repeatedly in the results and discussion sections with Figure 3 and the detailed municipality-level summaries in the supplement giving precise figures.

***1.2.3.** The cost targets are very conservative in requiring cost-neutrality from a health system perspective (i.e. implementation cost ≤ direct medical costs + outbreak vector control costs averted) and assuming only 3-5 years of benefits for the preferred TPP. Although a scenario of 10 years of benefits is also included in Table 2, the authors present the cost target of $2.33 as the minimum TPP, based on a scenario of achieving a 25% global burden reduction and only 5 years of benefit, with a PPC cost target of $0.24 (25% national burden reduction in all countries and only 3 years of benefit). These durations of benefit are too pessimistic given the evidence from field studies of Wolbachia durability in Ae. aegypti populations for at least 10 years.*

**1.2.3.** It is worth reiterating that these estimates should not be interpreted as predictions of the likely costs and benefits of *Wolbachia* programmes, but as a proxy for willingness to pay for *Wolbachia* implementation. We agree that there is good evidence that *Wolbachia* persists for at least 10 years and specifically included a scenario with 10 years of benefits in our analysis because of this (Table 2). However, these longer duration benefits were judged by members of the TAG (which included programme managers) as being less relevant when estimating willingness to pay for a new programme and hence why the 5-year option was chosen for the minimum criteria.

***1.2.4a.** The entomological modelling of the hybrid “suppress then replace” is based largely on theoretical estimates of suppression efficacy (Figure 4), or extrapolation of population suppression results from small scale pilot interventions. This reviewer questions whether the efficacy of these suppression methods can ever be achieved at the scale required to avert 25% of the global dengue burden. The assumptions around the suppressive effects of a single round of intervention seem overly simplistic, and are generally not consistent with field implementations that take many rounds of intervention to produce consistent and reproducible suppression (see Supplementary Table 1 references below). “With reductions in adult mosquito population size in the range of 8.18 – 43.51%, from a single application all methods of suppression were predicted to remove the ~1–10% mosquito population exacerbation seen in replacement only programmes.” (Page 12) Further, “Our model predicts that a prior suppression programme of five weekly rounds could reduce the number of Wolbachia mosquitoes required to reach target coverage within 6 months by 16-81% depending on suppression method used.” (Pages 12-13)*

**1.2.4a.** We agree that the evidence base for hybrid suppress and replace strategies is not as developed as that for *Wolbachia* replacement alone. It is this very fact that led WHO to develop a TPP for replacement but only a PPC for hybrid strategies. PPCs are not subject to the same specific criteria (like the 25% mentioned by the reviewer) as TPPs and come with clear guidelines that they should be tested using a range of methods and experimental designs before a TPP can be developed. We believe our modelling work is proportionate to the differing needs of these two distinct policy objectives.

 We also agree that achieving the efficacy of the hybrid suppress-then-release scenarios at scale would be challenging and generally only an option that might be considered if cost, community acceptability or speed considerations could not be met with a replacement-only approach. We state this explicitly in the relevant section of the results:

*“This may mean that rather than hybrid approaches superseding replacement-only approaches, their use may be restricted to areas where replacement-only cannot meet speed, acceptability, and cost goals.”*

 We also agree that it takes time and multiple rounds of application to consistently achieve suppression. Areas most likely to consider hybrid programmes will likely already have a large suppression component to their vector control programmes, so a hybrid programme could be considered more of an extension (e.g. continuing suppression for longer or at times of the year when suppression is limited) than a new application. Under such circumstances experienced personnel with a high degree of familiarity with the local vector ecology, supported by existing programme infrastructure would be more likely to achieve the levels of vector control suggested by these studies than if starting from scratch. Because we agree that it is difficult to make comparisons between different study designs for suppression effectiveness we also include fixed efficacies that readers can use instead that give a more standardised comparison between different suppression methods and can be used to explore lower effectiveness suppression options if they believe these are more realistic in their own contexts:

*“Additionally, suppression efficacy is highly context-dependent; to mitigate this we modelled an hypothetical 20%, 50%, and 80% suppression efficacy to allow clearer comparison between suppression techniques.”*

***1.2.4b.** Based on empirical data from a Male Wolbachia release program that targeted Ae. aegypti, there is questionable evidence that a 5-week suppression program would substantially impact mosquito population abundance. In the Supplementary Table 1, it indicates a 5-week suppression efficacy of 60%, and 10-week suppression efficacy of 90% (sourced from Ching et al. 2021). This is incorrect. Figure 2, Panel G indicates <10% suppression out to week 7 in the Yishun Core Area where releases were concentrated. In the Tampines Core area, suppression efficacy varied significantly over the first 12-13 weeks, ranging from 0-40%. Ching et al. (2021) presented a statistical analysis of the suppression effect (Figure 3, Panels A and C) and found no significant suppression in Yishun until the fourth month of releases, and variable results in Tampines (statistically significant suppression in first month, but not significant in the second month of releases).*

**1.2.4b.** We acknowledge the reviewers concerns regarding the source for the literature-derived efficacy values for Male *Wolbachia* release, which we cite as Ching (2021). Having revisited the paper and our extraction process we agree that the time interval over which these levels of suppression can be achieved is overoptimistic in this example. We have since searched for alternative studies that give more appropriate estimates of short-term effectiveness. The analyses have been updated to include literature-derived efficacy values for Male *Wolbachia* release sourced from Crawford et al (2020), a field trial which reported suppression efficacy values reaching approximately 65% and 92% at 5- and 10-weeks, respectively; values extracted using WebPlotDigitizer (Rohatgi, 2021) from Figure 6d. The previous analyses used 60% at 5-weeks and 95% 10-weeks and this update has only caused small changes in the population dynamics and have not altered the overall trends or our findings. In addition, Male *Wolbachia* values have been updated in Figures 4, 5, and 6, added to Supplementary Figure 2, and the literature source for Male *Wolbachia*release has been updated in Supplementary Table 1.

***1.2.4c.** Similarly for the “1gSLT” suppression rates of 45% and 75% at weeks 5 and 10, respectively (Supplementary Table 1. Suppression Efficacy Carvalho et al. 2015). These data were extracted from Figure 2, panel D, from the 5- and 10-week timepoints in release area A, the authors have failed to state that there were 7-months of releases undertaken across these combined Areas A and B prior to the 5- and 10-week suppression period over which the above rates were calculated (for Area A). Also of note was the very small size of the suppression area in this trial (Area A = 0.055km2). Again, the assumptions drawn from these studies are inaccurate and should be withdrawn.*

**1.2.4c.** We acknowledge the reviewers concerns that the literature-derived value extracted for 1gSLT was from the period of releases in Area A only (Carvalho et al, 2015), while previously there were seven months of releases across the combined Areas A and B. However, the prior suppression efforts concerned the refinement and scale up of technology and did not consistently impact vector populations until after the effort to area ratio was increased. This is confirmed within the paper: “﻿Up to 11th February 2012 we released into areas A and B (Fig 1), comprising 11 ha in total. However, despite improvements in rearing over the period, in this highly infested area we were unable to produce enough OX513A males with the available resources to consistently maintain a mating fraction of 50%, as judged by the percentage of fluorescent larvae. We therefore reduced the release area to an area of 5.5 ha (Fig 1A). As expected, the fluorescence ratio increased correspondingly” and further supported by the observations from Figure 2, panel B, that the suppression effort was increased approximately 3-fold from levels prior to release in Area A only. Since these hybrid scenarios are most informative to circumstances where suppression technology is already in use as part of the vector control programme, it is appropriate for us to assume the region already has the necessary resources for suppression and therefore use values from more mature examples where initial training and testing phases are omitted. This makes our assumptions about using such methods for hybrid programmes more comparable with our assumptions about replacement, for which we also assume relatively rapid implementations can be achieved (< 6 months) when compared to the currently published literature based on the same argument of product maturity and familiarity. Furthermore, the extracted values for this technology used in the analysis are conservative in the short-term when compared with another field trial reported by Harris *et al* (2012), which shows an estimated 82% and 68% efficacy after 5- and 10-weeks of intervention, respectively (extracted from Figure 2c). Finally, we agree that the size of the suppression area is not extensive, and this detail has been added to the Supplementary Table 1:

*“Extracted from Figure 2, panel D, from the 5- and 10-week timepoints of release in area A only, a concentrated area of 0.055km ^2^.”*

***1.2.4d. **How have the SIT suppression efficacy estimates been validated? In Supplementary Table 1, it states there is no currently published trials for suppression using SIT, and also in the Methods it states “There are currently no published randomised controlled trials for suppression of Aedes mosquitoes using SIT, therefore 50% efficacy is shown for SIT in analyses where only literature-derived efficacy is used. What is the “literature derived efficacy”?*

**1.2.4d.** Previously, we had no literature reference for SIT and therefore this statement from the manuscript refers to the fact that an hypothetical 50% efficacy value was used instead of a literature-derived efficacy in figures which otherwise presented only literature-derived efficacy values; this was stated in the legend. However, the lack of a SIT literature source has now been highlighted as an oversight by the other reviewer and a literature-derived efficacy of approximately 49% at 5-weeks and 77% at 10-weeks has been extracted from the field trial by De Castro Poncio et al (2021), Figure 2b. All analyses which previously used an hypothetical 50% efficacy for this suppression technique have been updated to use the literature-derived 49% efficacy, correspondingly there has been only marginal changes in resulting population dynamics and no change in the overall trends or findings discussed. The methods section for SIT suppression have been updated to include this development:

*“Parameters for the hypothetical fixed rate efficacy of 20%, 50%, and 80% were calculated by comparing the total adult population at model equilibrium with the minimum adult population reached after five weeks of application. The literature-derived efficacy values were 49% five weeks after the last suppression period and 77% ten weeks after the last suppression period (33), calculated by comparing the total adult population at model equilibrium to the total adult population after five- or ten-weeks of suppression which achieved the desired efficacy (summarised in Supplementary Table 1). A caveat of this approach is that the resulting minimum adult population is reached later than five- or ten-weeks, therefore, the maximum efficacy calculated in these scenarios is marginally greater than the literature value stated (shown in Supplementary Figure 2).”*

 In addition, SIT values have been updated in Figures 4, 5, and 6, added to Supplementary Figure 2, and the SIT literature source has been added to Supplementary Table 1.

***1.2.4e.** The assumptions underpinning the modelling of the suppress then replace section of the paper appear to be erroneous, or at least very optimistic of their effectiveness in terms of suppression of the mosquito populations. This section is highly speculative, adds little to the paper and should be removed.*

**1.2.4e.** We acknowledge the reviewer’s concerns about the measurement and comparability of effectiveness of suppression-based vector control for Aedes mosquitoes and agree that the values we use from the cited studies are context and application specific (and state so in the discussion). WHO is currently conducting a Cochrane review of *Aedes* mosquito control effectiveness that originally planned to inform these parameters with a more rigorous evidence base, however delays meant that we instead chose values based on a single well-designed study. Acknowledging this approach may introduce bias, we also simulate a range of fixed values for suppression effectiveness (20%, 50%, and 80%) which allowed us to better compare different suppression methods for the purpose of hybrid programmes and set expectations for areas where suppression efficacy is likely to be lower. We hope that the above improvements to the evidence we cite and further explanation of the differing objective of the modelling for the TPP and PPC more clearly clarifies the value of this section of the work.

***1.2.5.** “All hybrid programmes had the potential to offer cost savings over replacement alone, depending on the cost of suppression” (Page 14). This statement is not supported by presentation of any costing data for suppression. From an economic perspective, there is no evidence that a hybrid approach of suppression followed by replacement would be more cost effective than Wolbachia replacement alone, and a recent study based on data from Singapore’s Wolbachia suppression program suggests that a hybrid suppression-followed-by-replacement program could be significantly more expensive (Soh et al. 20211). The current annual per capita cost of suppression in Singapore is approximately USD $5 per person reached. This cost is roughly equivalent to the one-time, all-inclusive cost required to achieve Wolbachia replacement across some target settings (Brady et al BMC Med 2020; Turner et al PLoS Negl Trop Dis 2023). The ‘potential cost savings enabled by a hybrid approach’ presented in Table 3 “assumes suppression has no cost or is an in-kind contribution”, which is entirely implausible in any setting, let alone in a scenario of large-scale global implementation across the 73 countries (>900,000 km2), which would be required to achieve the TPP minimum targets. Without a more extensive analysis of the efficacy of suppression programs and their cost, the current analyses are highly speculative and add little to the paper. This reviewer feels the whole section on hybrid “suppress then replace” should be removed.*

**1.2.5.** As mentioned above, the hybrid suppress-then-replace scenarios are specifically for circumstances where suppression is already part of an existing vector control programme. Since areas which would benefit from *Wolbachia* replacement are likely to already engage in vector control we included this model to quantify the potential gains from utilising existing suppression resources when transitioning to novel *Wolbachia* technology. In these specific contexts, suppression resources are likely to already have been purchased therefore the cost of suppression is assumed to be zero, however if this were not the case then the additional cost savings from implementing a new suppression technique are likely to be marginal. This has been further emphasised in the manuscript:

*“…then a hybrid programme will cost less than replacement alone. This might refer to specific circumstances when suppression might be achieved at negligible cost, for example, if it’s already part of a vector control programme. These are ideal situations where the resources for suppression are already in place, however, in most cases the potential savings achieved by a hybrid suppress then replace programme won’t justify the additional cost of implementing a novel suppression programme.”*

 We acknowledge the reviewers concern regarding the cost of suppression, and our conclusions around cost savings previously recognise this depends on the cost of suppression, for example in the results:

*”All hybrid programmes had the potential to offer cost savings over replacement alone, depending on the cost of suppression… This, however, does not take into account the costs of suppression. A different interpretation of these results would be: if suppression can be achieved for less than these costs, then a hybrid programme will cost less than replacement alone.”*

 And in the discussion:

*“We predict that insect-based suppression methods (1gSLT, SIT, Male Wolbachia release) will be more effective than conventional suppression tools, but also outline a limited cost window which may be challenging for insect-based suppression methods to achieve. Investment in new infrastructure to conduct insect-based suppression may not be justified for a one-off suppression, but between overlapping resource requirements for suppression and replacement, ongoing use post-replacement (e.g., outbreak control or to achieve dengue elimination) and a continued drive to lower costs of mosquito suppression (51), this investment cost may be justified.”*

 Finally, it is worth repeating that the aim of the PPC is to motivate the development of new products and strategies. It is precisely because one-off suppression campaigns can’t meet these cost objectives that a PPC is needed, and these results give approximate targets that novel products or strategies could aim for to be useful for a hybrid suppress and replace approach.

**1.3. Minor comments:**

***1.3.1.** Page 3, Introduction, second paragraph: The virus-blocking phenotype is specific to the species of Wolbachia, mosquito, and virus - the authors should use more specific language here.*

**1.3.1.** We agree that more specific language should be used when referring to the virus-blocking potential of *wMel*, therefore this has been clarified in text:

*“Ae. aegypti mosquitoes infected* *with certain strains of the bacterium Wolbachia, such as wMel, wMelPop, and wAlbB (7)* *show reduced rates of virus dissemination, making them less capable of transmitting arboviruses (8).”*

***1.3.2.** Page 3, Introduction, second paragraph: There is no empirical evidence that temporary increases in the Ae. aegypti mosquito population have been a key barrier to community acceptability of Wolbachia releases; this should be expressed as theoretical consideration.*

**1.3.2.** We agree that temporary increases in *Ae. aegypti* mosquito population have not been a barrier to community acceptability in the past, therefore we have edited the text to reflect this nuance:

*“All of these options increase cost and can also lead to undesirable temporary increases in the Ae. aegypti mosquito population which should be addressed during community engagement to avoid it becoming a barrier to community acceptability (9, 10).”*

***1.3.3.** Page 6, Results, paragraph 1 (and throughout): the terminology ‘coverage’ is misleading as it implies the % coverage of a geographic area, whereas the authors are referring to % Wolbachia prevalence in the local Ae. aegypti population. The proportion of Wolbachia-infected mosquitoes in the population should be considered the “percentage prevalence,” not the target coverage.*

**1.3.3.** We agree the terminology “coverage” could be a potential point of confusion, therefore we have clarified the phrasing of coverage in the introduction so that the usage is clear throughout:

*“…self-sustaining coverage, defined as the percentage of Ae. aegypti population infected with Wolbachia, can be achieved by: increasing the number of releases…”*

 Our definition of coverage must be consistent with that used by the TAG for the TPP and was hence a decision outside of our control.

***1.3.4.** Page 11, Figure 3: The axes titles and figure legend needs to make explicit what time period this data relates to, in terms of the cumulative cases and costs averted. Presumably 5 years of benefit, since the $2.33 cost threshold is shown, however this is inconsistent with the $0.24 preferred criteria referred to in the figure legend, which relates to 3 years of benefit. And the lower horizontal dotted line appears to be at $0.39, not $0.24.*

**1.3.4. **We thank the reviewer for their feedback and have changed the axis labels and figure legend to make it clear what the temporal denominator is for each measure.

***1.3.5.** Page 16, Discussion: “Cost continues to be a barrier to wider adoption of Wolbachia replacement when its high costs but long-term benefits are compared to lower cost but short acting suppression methods”. This statement needs to reflect the fact that Wolbachia replacement has an evidence base for epidemiological impact while the short acting suppression methods do not.*

**1.3.5.** Agreed, this sentence now reads:

*“Cost continues to be a barrier to wider adoption of Wolbachia replacement when its high costs but long-term benefits are compared to lower cost but short acting suppression methods, despite differences in the evidence base underpinning these benefits”*

**References**

 Carvalho, D. O., McKemey, A. R., Garziera, L., Lacroix, R., Donnelly, C. A., Alphey, L., Malavasi, A., & Capurro, M. L. (2015). Suppression of a field population of Aedes aegypti in Brazil by sustained release of transgenic male mosquitoes. *PLoS Neglected Tropical Diseases*, *9*(7), 1–15. https://doi.org/10.1371/journal.pntd.0003864

 Ching, N. L. (2021). *Wolbachia-mediated sterility suppresses Aedes aegypti populations in the urban tropics*. https://doi.org/10.1101/2021.06.16.21257922

 Crawford, J. E., Clarke, D. W., Criswell, V., Desnoyer, M., Cornel, D., Deegan, B., Gong, K., Hopkins, K. C., Howell, P., Hyde, J. S., Livni, J., Behling, C., Benza, R., Chen, W., Dobson, K. L., Eldershaw, C., Greeley, D., Han, Y., Hughes, B., … White, B. J. (2020). Efficient production of male Wolbachia-infected Aedes aegypti mosquitoes enables large-scale suppression of wild populations. *Nature Biotechnology*, *38*(4), 482–492. https://doi.org/10.1038/s41587-020-0471-x

 De Castro Poncio, L., Dos Anjos, F. A., De Oliveira, D. A., Rebechi, D., De Oliveira, R. N., Chitolina, R. F., Fermino, M. L., Bernardes, L. G., Guimarães, D., Lemos, P. A., Silva, M. N. E., Silvestre, R. G. M., Bernardes, E. S., & Paldi, N. (2021). Novel Sterile Insect Technology Program Results in Suppression of a Field Mosquito Population and Subsequently to Reduced Incidence of Dengue. *Journal of Infectious Diseases*, *224*(6), 1005–1014. https://doi.org/10.1093/infdis/jiab049

 Rohatgi, A. (2021). *WebPlotDigitizer V4.5*. https://automeris.io/WebPlotDigitizer
